# Identifying a Terminal Nickel–Oxygen Complex Bearing an Unsymmetrical β‑Diketiminate Ligand

**DOI:** 10.1021/jacsau.6c00356

**Published:** 2026-05-08

**Authors:** Si-Hong Chen, Tzu-Hsien Yang, Yu-Lun Chang, Wei-Syuan Lin, Chuan-Hung Chuang, Cheng-Han Yang, Hsing-Yin Chen, Ming-Li Tsai, Sodio C. N. Hsu

**Affiliations:** † Department of Medicinal and Applied Chemistry, Drug Development and Value Creation Research Center, College of Life Sciences, 38023 Kaohsiung Medical University, Kaohsiung 80708, Taiwan; ‡ Department of Chemistry, 34874 National Sun Yat-Sen University, Kaohsiung 80459, Taiwan

**Keywords:** β-Diketiminate, Terminal Ni−O, Chelation, Unsymmetrical, Nickel-oxyl, Hydrogen-atom abstraction

## Abstract

Nickel–oxygen species are proposed as key intermediates in various biological nickel-containing catalytic and enzymatic processes. We report the successful synthesis and crystallographic characterization of the first terminal nickel–oxygen complex featuring a tridentate *N*-aryl-*N*′-methylpyridyl β-diketiminate ligand. This species can be formally classified as either a nickel-oxo (Ni^III^=O) or nickel-oxyl (Ni^II^–O^•^) species, exhibiting dual bonding characteristics. The experimental observations and DFT calculations suggest the bonding is best described as a resonance hybrid of Ni^III^=O and Ni^II^–O^•^ states, with the Ni^II^–O^•^ form likely predominant.

## Graphical abstract

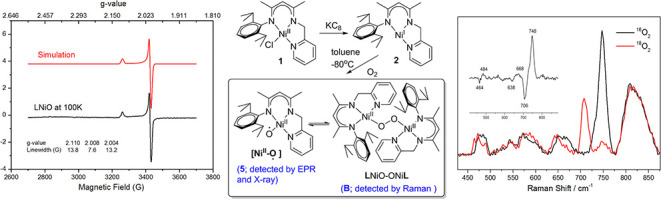

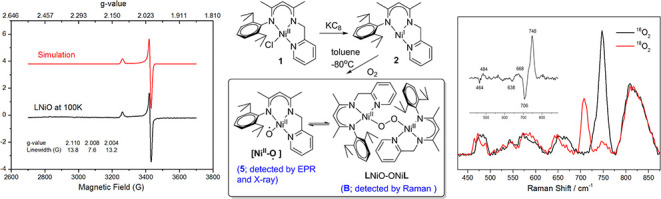

Transition metal complexes featuring terminal oxo or oxyl ligation are proposed as key intermediates in numerous biological and abiological catalytic oxidation processes.^1–10^ The isolation and synthesis of metal-oxo or -oxyl species could enhance our understanding of bioinspired chemistry. Nickel-oxo and nickel-oxyl species have attracted considerable attention because they have garnered significant interest due to their potential as intermediates in nickel-mediated oxidation reactions.^11–18^ For example, a [Ni^III^-oxo] species was proposed as the active oxidant in the [Ni^II^(TPA)]^2+^ [TPA = tris­(2-pyridylmethyl)­amine] catalyzed alkane hydroxylation.^14,15^ Driess and co-workers suggested that a transient nickel–oxygen species [L^*i*Pr^Ni = O ↔ L^*i*Pr^Ni–O^•^] (L^*i*Pr^ = HC­(CMeNC_6_H_3_^*i*^Pr_2_)_2_) is generated through N_2_O monooxygenation of [(L^*i*Pr^Ni^I^)_2_(μ-η^3^:η^3^-C_6_H_5_Me)], acting as a hydrogen scavenger and subsequently formed an unusual [(L^*i*Pr^Ni^II^)_2_(μ–OH)_2_].^19^ Additionally, experimental and theoretical studies propose a nickel–oxygen species as an effective oxidant for the challenging gas-phase conversion of methane to methanol.^20–22^ Despite this, spectroscopic investigations of nickel–oxygen species remain limited, and no crystallographic characterization exists.^23–26^

Drawing inspiration from symmetrical β-diketiminato nickel­(I) complexes^27–32^ and prior studies on unsymmetrical β-diketiminato systems,^33–36^ we propose that the *N*-aryl-*N*′-methylpyridyl β-diketiminate ligand (**L**H) acts as a hemilabile chelator. The flexible methylpyridyl arm provides electronic tunability and enables reversible coordination, allowing the ligand to adapt its denticity and stabilize high-valent or radicaloid nickel–oxygen units. This design creates an unsymmetric donor environment that promotes spin delocalization onto the oxygen atom, disfavors bimolecular decomposition, and facilitates reorganization from the T-shaped Ni­(I) precursor (**2**) to a four-coordinate terminal Ni–O species (**5**). Accordingly, we report the crystallographic characterization of the first terminal nickel–oxygen complex supported by this tridentate ligand framework (Scheme 1).

**Scheme 1:**
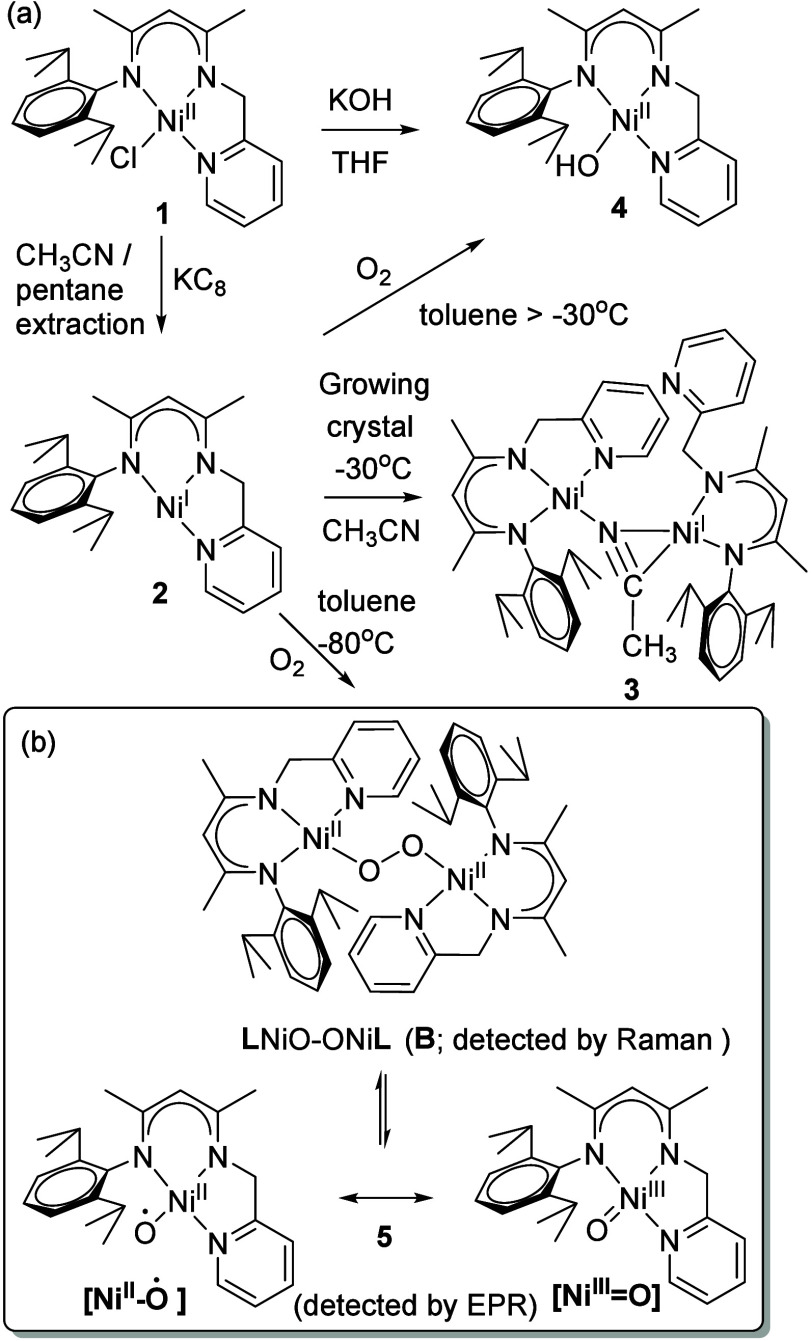
^a^

a (a) Synthesis of the *N*-aryl-*N*′-methylpyridyl β-diketiminato nickel complexes. (b) Formation of **5** as a Ni^III^=O or Ni^II^–O^•^ species and its dinickel-peroxo species.

Deprotonation of LH with KO^t^Bu in the presence of [NiCl_2_(2,4-lutidine)_2_] afforded **L**Ni^II^Cl (**1**), which was reduced with KC_8_ to give the paramagnetic T-shaped Ni­(I) complex **L**Ni^I^ (**2**). Both complexes have been independently reported,^37,38^ our data are fully consistent with the literature. Complex **1** is diamagnetic and air-stable, while **2** is highly air- and moisture-sensitive, exhibiting a rhombic signal (*g*_*1*_ *=* 2.288, *g*_2_ = 2.134, and *g*_3_ = 2.035) with ^14^N superhyperfine coupling (A_N_ = 17 G) to one ligand nitrogen and higher spin density on N2 (Figure 1, Table S3).^39–41^

**Figure 1:**
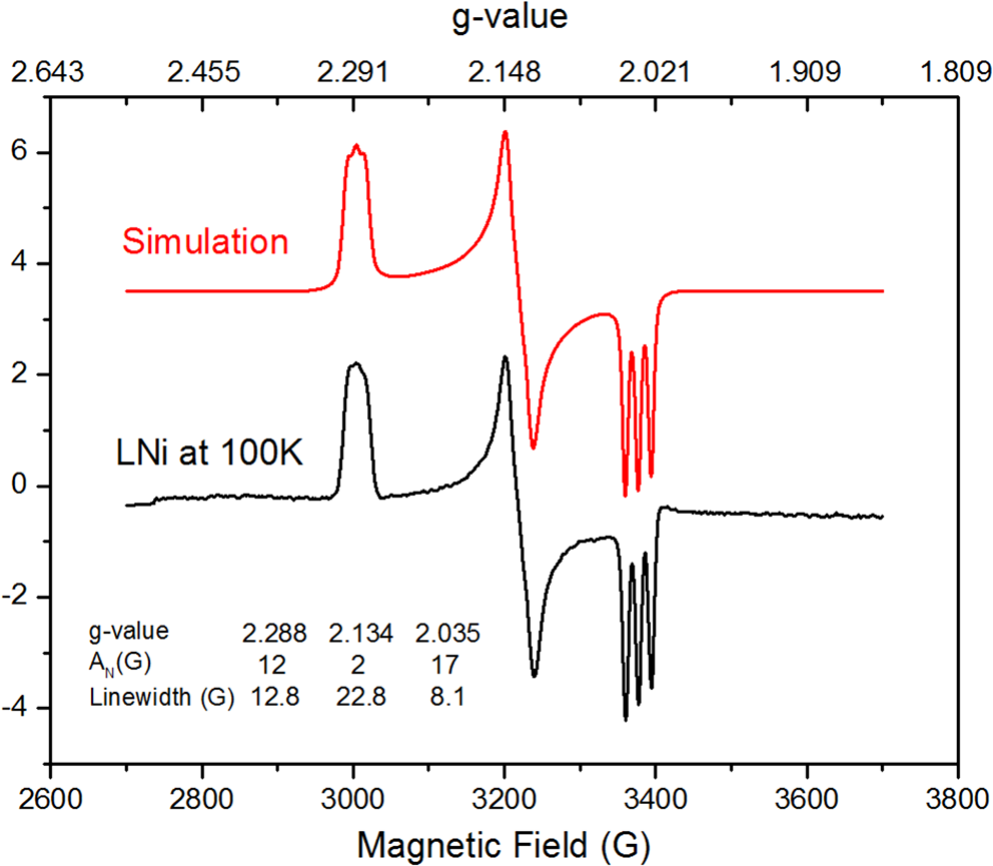
X-Band EPR spectra of **2** in toluene at 100 K.

Crystallization of **2** from pentane or hexane revealed the expected T-shaped geometry (Figure 2a). Interestingly, crystallization from acetonitrile gave an unexpected dinickel­(I) species, **3**, where acetonitrile bridges two nickel centers in a μ-κ^1^:η^2^-bonding mode (Figure 2b). Notably, the N≡C bond length of 1.236(7) Å reflects back-bonding from the η^2^-bound nickel­(I) center, longer than the 1.157(9) Å in free acetonitrile.^42^ The μ-κ^1^:η^2^-bound nitrile has been observed with only selected transition metals^43^ and has only been observed for nickel(0) species.^44,45^

**Figure 2:**
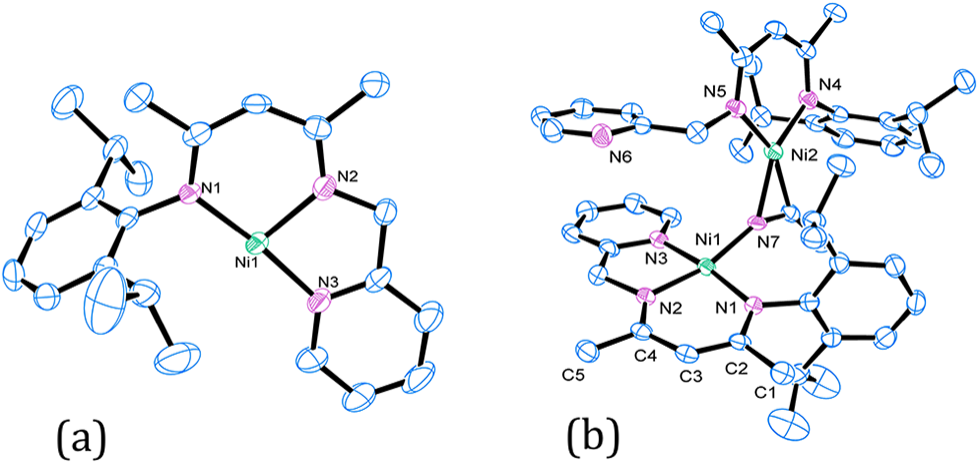
Molecular structure of (a) **2** and (b) **3**. Thermal ellipsoids are drawn at a 50% probability level. Hydrogen atoms are omitted for clarity.

Exposure of a toluene solution of **2** to dry dioxygen at ambient temperature triggered an immediate color shift from deep red to green, signaling the formation of the nickel­(II)-hydroxyl species **L**Ni^II^–OH (**4**). Fractional crystallization from the reaction mixture yielded green crystals of **4** in low yield (∼25%). The crystal structure, as shown in Figure S5, reveals a low-spin square-planar Ni­(II) center with a terminal –OH group. The ^1^H NMR spectra exhibit diagnostic peaks for the terminal hydroxyl (−4.73 ppm) and the *N*-aryl-*N*′-methylpyridyl β-diketiminate ligand framework (Figure S6). The Ni–O distance in **4** (1.854(2) Å) aligns with those of similar terminal nickel­(II)-hydroxyl square-planar complexes.^46–49^ The solid-state FTIR spectrum of isolated **L**Ni^II^–OH (**4**) shows a distinct O–H stretch at ν­(O–H) = 3637 cm^–1^, shifted to ν­(O–D) = 2633 cm^–1^ upon exposure to CH_3_OD (Figure S7). Additionally, complex **4** can be prepared in a higher yield (82%) via salt metathesis from the reaction of **L**Ni^II^Cl (**1**) with KOH.

A “side-on” superoxo-nickel complex, [L^*i*Pr^Ni^III^(O_2_)], with a symmetrical β-diketiminato ligand, was previously reported from the reaction of [(L^*i*Pr^Ni^I^)_2_(μ-η^3^:η^3^-C_6_H_5_Me)] with dry dioxygen at −78 °C.^19^ This finding motivated us to reexamine the reaction of **2** with dry dioxygen at a lower temperature, in contrast to the room temperature reaction that produced **4**. Spectrophotometric titration of **2** with dioxygen at −80 °C showed a 2:1 Ni/O_2_ ratio in spectral changes (Figures 3 and S8), suggesting the possible formation of a nickel-monooxygen species **5** or dinickel–peroxo species under these conditions.

**Figure 3:**
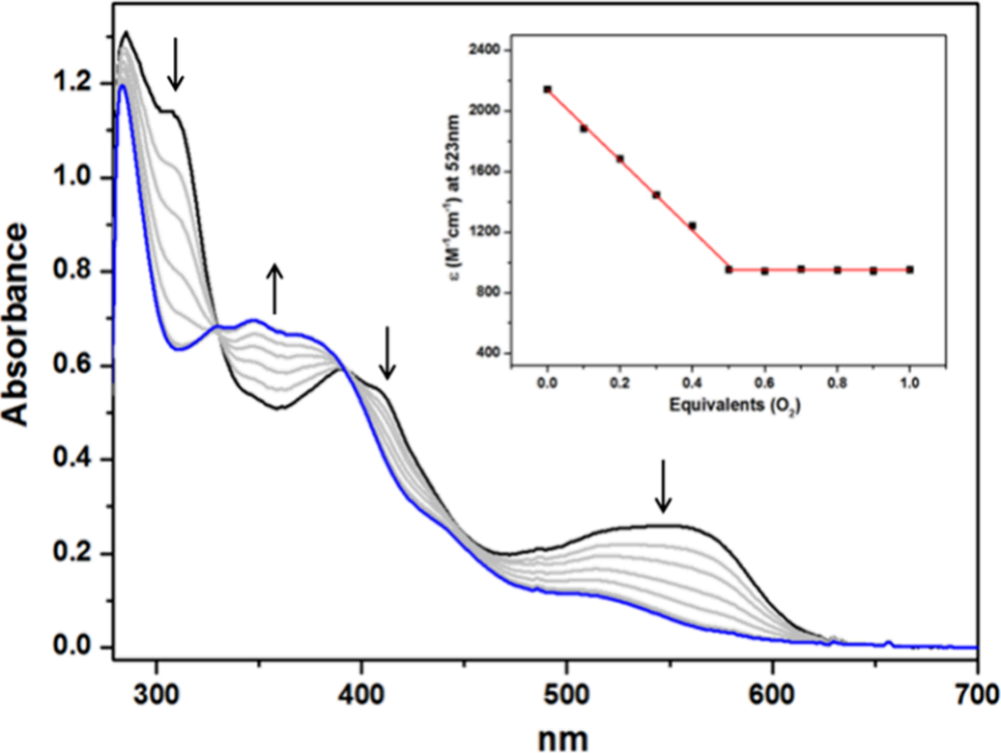
UV–vis changes accompanying oxygenation of **2** in toluene at −80 °C, using starting concentrations of 0.12 mM. The 0.1 equiv of oxygen was added at a rate of 2 per minute until 1.0 equiv was reached. The initial and final spectra are represented in black and blue, respectively. The insets show the results of spectrophotometric titrations of the complex **2** with O_2_ at 523 nm, plotted as linear fits at a 2:1 Ni/O_2_ ratio.

To explore intermediates formed during the oxygenation of **2**, low-temperature Raman spectroscopy was performed by exposing a frozen solution of **2** to dioxygen at −80 °C. The resulting spectra exhibited three O_2_ isotope-sensitive bands at 484, 668, and 748 cm^–1^, which shifted to 464, 638, and 706 cm^–1^ with ^18^O_2_ substitution (Figure 4). These isotopic shifts, with ratios of ν­(Ni–^16^O)/ν­(Ni–^18^O) = 1.041 and ν­(^16^O–^16^O)/ν­(^18^O–^18^O) = 1.060, are assigned to Ni–O and O–O stretching vibrations, respectively, aligning with previously reported nickel–oxygen species.^50,51^ DFT calculations further support this assignment, identifying the 668 cm^–1^ band (theor. value 684 cm^–1^) to Ni–O bond vibrations of **L**Ni–O^•^ (**5**). The 484 cm^–1^ (theor. value 523 cm^–1^) and 748 cm^–1^ (theor. value 770 cm^–1^) bands may attribute to the Ni–O and O–O vibrations, respectively, of a peroxo-bridged dinickel intermediate, **L**Ni–O–O–Ni**L** (**B**, see Figure S9).

**Figure 4:**
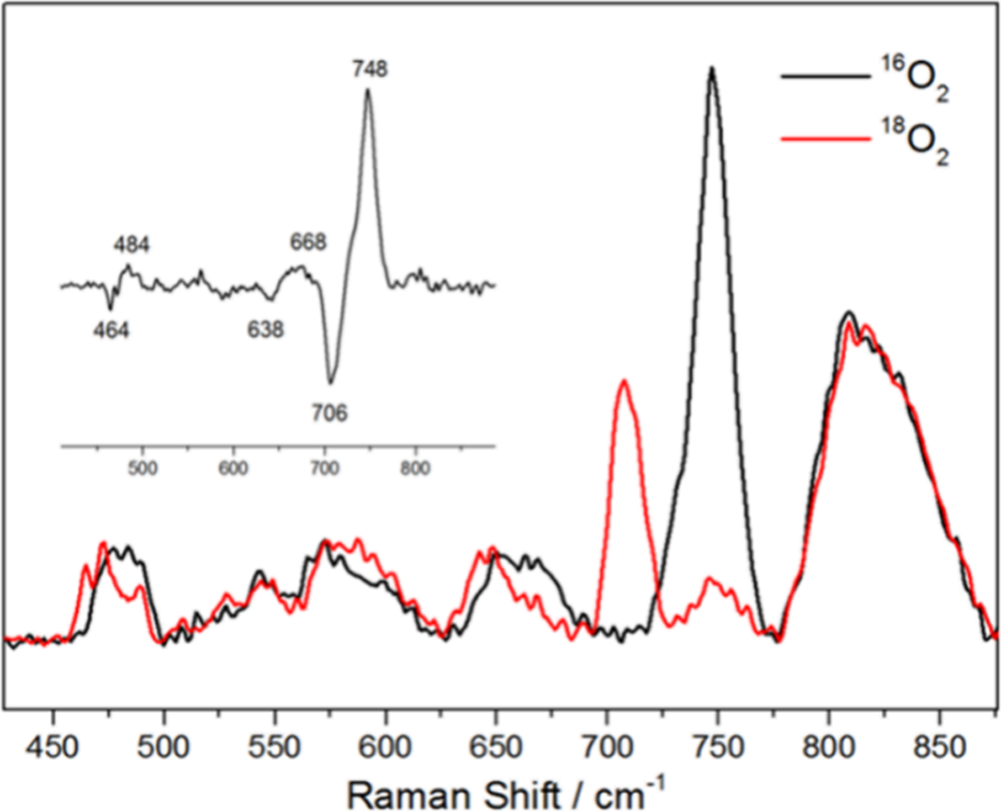
Raman spectra (λ_ex_ = 532 nm) of complex **2** recorded in MTHF at −80 °C prepared with ^16^O_2_ (black) and partially ^18^O_2_ labeled (red); inset: difference spectrum.

To further probe the reaction pathway, UV–vis absorption spectra of **2** with dry dioxygen were monitored at 523 nm at 10-s intervals at −110 °C, revealing transient intermediate species during the conversion to **5** (Figure S10). Based on these data together with low-temperature Raman spectroscopy, we propose a three-step mechanism for the formation of **5** (Scheme S1): (i) initial binding of O_2_ to **2** to form the Ni-η^1^-superoxo species **L**Ni^II^–O_2_^•–^ (**A**); (ii) rapid dimerization of **A** with a second equivalent of **2** to form the Ni_2_-μ-1,2-peroxo species **L**Ni^II^–O_2_^2–^–Ni^II^ **L** (**B**); and (iii) O–O bond homolysis of **B** to afford the nickel-monooxygen species **5** (Figure S9). DFT calculations indicate that the formation of **A** and **B** is thermodynamically favorable, and their binding modes are consistent with literature precedents.^51–53^ The absence of superoxo vibrational features in the Raman spectra supports rapid conversion of **A** to **B**, underscoring the dynamic nature of the low-temperature oxygenation process.

A light-red crystal of **5** was obtained from the reaction mixture stored at −40 °C. The molecular structure of **5** reveals a square-planar nickel center bound by an oxygen atom and a tridentate *N*-aryl-*N*′-methylpyridyl β-diketiminate ligand (Figure 5). The Ni–O distance in **5** (1.750(4) Å) is shorter than the Ni–OH bond length in **4** (1.854(2) Å) and bis­(μ-oxo)­dinickel­(III) complexes (∼1.85(2) Å).^54–56^ Additionally, it is shorter than the Ni–O distances in previously reported Ni^II^_2_(μ-1,2-peroxo)­(1.834­(2) Å)^51^ and Ni^III^_2_(μ-1,2-peroxo)­(1.796­(9) Å)^52^ complexes. These comparisons indicate that **5** likely contains a terminal nickel­(III)-oxo or nickel­(II)-oxyl species.

**Figure 5:**
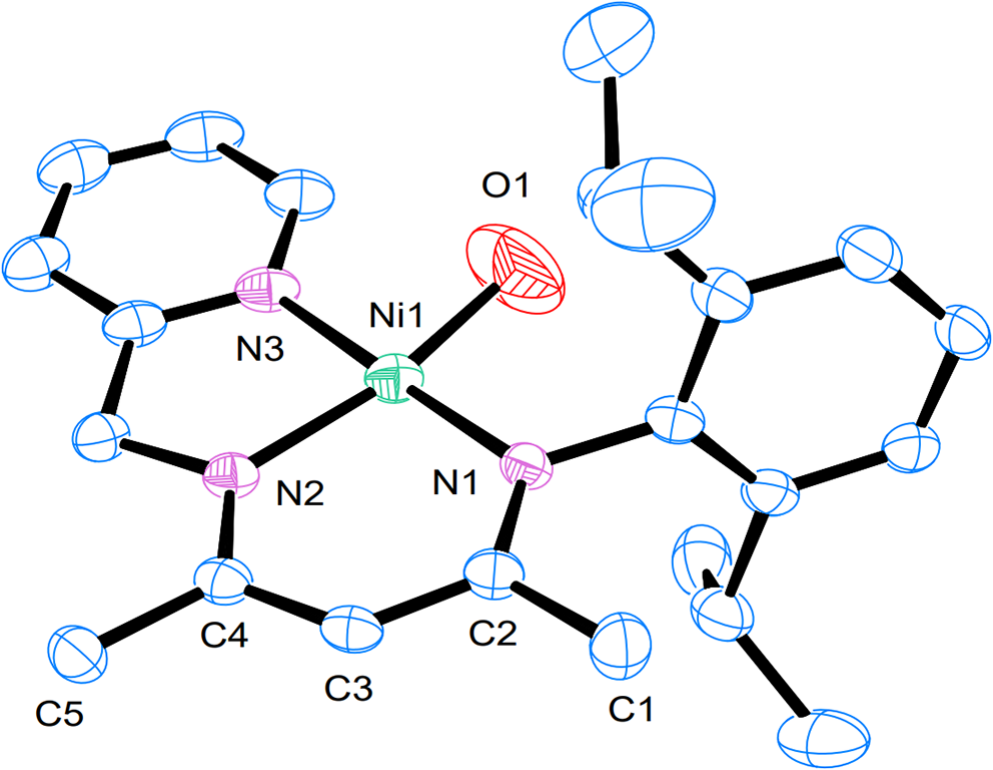
Molecular structure of **5**. Thermal ellipsoids are drawn at a 50% probability level. Hydrogen atoms are omitted for clarity.

Complex **5** is stable at −80 °C but decomposes rapidly above −30 °C. Monitoring the thermal decay by UV–vis spectra indicated a nickel­(II)-hydroxyl species **4** formation, implying the hydrogen atom abstraction (HAA) reactivity of **5** (Figure S11). Variable-temperature EPR experiments confirmed this decay, with the characteristic signal of **5** diminishing above −30 °C (Figures S12–13). In-situ generation of **5** by bubbling O_2_ into a toluene solution of **2** at −80 °C afforded an axial X-band EPR spectrum (g_1_ = 2.110, g_2_ = 2.008, g_3_ = 2.004), well reproduced by simulation (Figure 6). Frontier molecular orbital analysis (Figure S17) reveals that the SOMO possesses significant d_*xz*_ character. Spin–orbit coupling with nearby filled d_*yz*_ (−4.72 eV) and d_z^2^_ (−4.92 eV) orbitals accounts for the axial anisotropy and g-value deviation from g_e_ = 2.0023. These results indicate that the characteristic axial EPR signal arises from low-lying excited states and the specific d-orbital composition of the Ni-centered radical. EPR spin quantification of in situ oxygenated **2** samples revealed 41.8% conversion to the EPR-active nickel-monooxygen species **5** (Figure S14). Combined with the Raman data, this indicates a 1:0.7 equilibrium ratio between species **5** (41.8%) and the peroxo-bridged dinickel intermediate **B** (an EPR-silent μ-1,2-peroxo dinickel species; 29.1%) at –80 °C, suggesting that the 2 **L**Ni^II^–O^•^ (or **L**Ni^III^=O) ⇌ **L**NiO–ONi**L** equilibrium is slightly biased toward the oxyl pathway at –80 °C.

**Figure 6:**
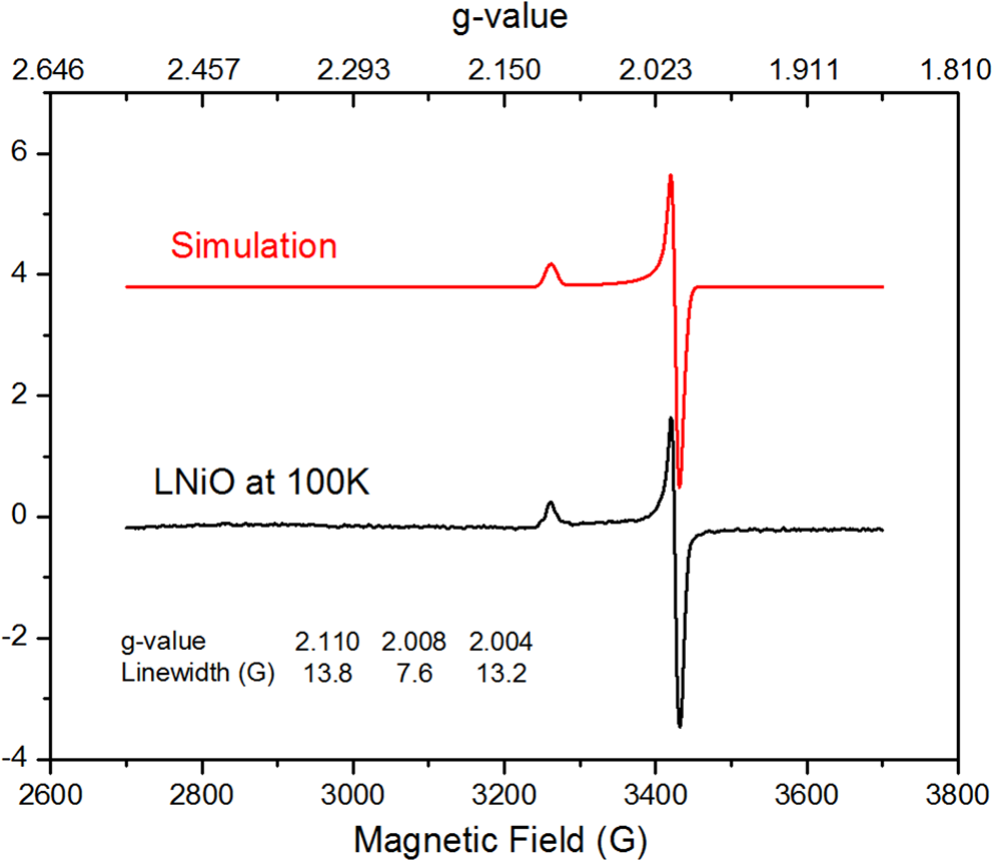
X-Band EPR spectra of *in situ* generated compound **5** in toluene at 100 K.

To investigate the mechanism of the oxidation pathway, N_2_O was used as an alternative oxygen-atom source. UV–vis titration and in situ X-band EPR spectra confirmed formation of the same nickel-monooxygen species **5** observed with O_2_ (Figure S15). Complementary resonance Raman measurements revealed the characteristic O–O stretching band at 748 cm^–1^ of the μ-1,2-peroxo dinickel intermediate **B** (**L**NiO–ONi**L**). The identical spectroscopic signatures obtained from both O_2_ and N_2_O indicate that the two oxidants converge on a common Ni–O intermediate. The simultaneous observation of **5** and **B** further supports a dynamic, reversible equilibrium between the terminal nickel–oxygen species and the peroxo-bridged dimer at low temperature.

To elucidate the bonding nature of the terminal nickel monooxygen species **5**, which can be formally classified as either **L**Ni^III^=O or **L**Ni^II^–O^•^, a molecular orbital (MO) analysis was conducted. As shown in Figure S17, MO93 corresponds to the σ-bonding interaction between the Ni d_x^2^–y^2^_ and O p_*x*_ orbitals, with additional contributions from the ligand nitrogen atoms. MO104 and MO106 represent the two π-bonding interactions, while MO112 and the SOMO (MO113) are the corresponding π* antibonding orbitals between Ni and O. Notably, the oxygen p_*z*_ orbital contributes substantially to the SOMO, indicating significant radical character on the terminal oxygen. As σ-bonding is stronger than π-bonding, MO93 lies lower in energy than the π-bonding MOs. Overall, six electrons occupy bonding orbitals (MO93, MO104, MO106) and three occupy antibonding orbitals (MO112 and SOMO113), affording a formal Ni–O bond order of 1.5. This was corroborated by natural population analysis (NPA), which indicated that the spin density is predominantly localized on the terminal oxygen (0.670 on O and 0.277 on Ni, Table S3). For more precise bond order data, Mayer, Wiberg, and Fuzzy bond order analyses were performed, and the values were 1.2, 1.6, and 1.8, respectively.^57^ Therefore, the bonding nature of **5** can be best described as a resonance hybrid of Ni^III^=O and Ni^II^–O^•^ states, with the Ni^II^–O^•^ form likely predominant.

To understand the chemical reactivity of **5**, stoichiometric reactions with PPh_3_ and TEMPOH were performed. After treatment of **5** with PPh_3_ at 183 K, followed by warming to room temperature, O=PPh_3_ was obtained in 30% yield, as determined by ^31^P NMR (Figure S18), indicating limited oxygen-atom transfer (OAT) reactivity. In contrast, the reaction of **5** with TEMPOH under identical conditions produced TEMPO radical and **L**Ni^II^–OH (**4**) in 45% isolated yield after recrystallization. However, EPR spin quantification of the TEMPO radical revealed a significantly higher conversion of ca. 76% (Figures S19). No reactions occurred between **4** and TEMPO (Figure S21). These studies demonstrate that complex **5** exhibits efficient hydrogen atom abstraction (HAA) reactivity toward TEMPOH, while its OAT reactivity toward PPh_3_ is comparatively limited. This dual reactivity profile is consistent with the resonance hybrid nature of the Ni^III^=O and Ni^II^–O^•^ states.

In summary, the *N*-aryl-*N*′-methylpyridyl β-diketiminate ligand framework stabilizes the nickel–oxygen unit, enabling the formation of a unique terminal nickel-monooxygen species **5** at low temperature. Based on the EPR data and DFT calculations, this paramagnetic nickel-monooxygen species **5** is best characterized as a resonance hybrid of Ni^III^=O and Ni^II^–O^•^ states with a planar tetracoordinate geometry. Additionally, low-temperature studies reveal a rapid equilibrium between the mononuclear oxyl **L**Ni–O^•^ and the peroxo-bridged dinickel intermediate **L**NiO–ONi**L**, highlighting the dynamic nature of the Ni–O system.

## Supplementary Material



## Notes

Additional figures, tables, and crystallographic data (PDF)

## References

[1] Larson V. A., Battistella B., Ray K., Lehnert N., Nam W. (2020). Iron and manganese
oxo complexes, oxo wall and beyond. Nat. Rev.
Chem..

[2] Shimoyama Y., Kojima T. (2019). Metal-Oxyl Species and Their Possible
Roles in Chemical
Oxidations. Inorg. Chem..

[3] Elwell C. E., Gagnon N. L., Neisen B. D., Dhar D., Spaeth A. D., Yee G. M., Tolman W. B. (2017). Copper-Oxygen
Complexes Revisited:
Structures, Spectroscopy, and Reactivity. Chem.
Rev..

[4] Oloo W. N., Que L. (2015). Bioinspired Nonheme Iron Catalysts
for C-H and C = C Bond Oxidation:
Insights into the Nature of the Metal-Based Oxidants. Acc. Chem. Res..

[5] Cook S. A., Borovik A. S. (2015). Molecular Designs
for Controlling the Local Environments
around Metal Ions. Acc. Chem. Res..

[6] Nam W. (2015). Synthetic
Mononuclear Nonheme Iron-Oxygen Intermediates. Acc. Chem. Res..

[7] Puri M., Que L. (2015). Toward the Synthesis
of More Reactive S = 2 Non-Heme Oxoiron­(IV)
Complexes. Acc. Chem. Res..

[8] Gagnon N., Tolman W. B. (2015). [CuO]^+^ and [CuOH]^2+^ Complexes:
Intermediates in Oxidation Catalysis?. Acc.
Chem. Res..

[9] Nam W., Lee Y.-M., Fukuzumi S. (2014). Tuning Reactivity and Mechanism in
Oxidation Reactions by Mononuclear Nonheme Iron­(IV)-Oxo Complexes. Acc. Chem. Res..

[10] Usharani D., Janardanan D., Li C., Shaik S. (2013). A Theory for Bioinorganic
Chemical Reactivity of Oxometal Complexes and Analogous Oxidants:
The Exchange and Orbital-Selection Rules. Acc.
Chem. Res..

[11] Corona T., Company A. (2016). Spectroscopically Characterized Synthetic Mononuclear
Nickel-Oxygen Species. Chem.—Eur. J..

[12] Yao S., Driess M. (2012). Lessons from
Isolable Nickel­(I) Precursor Complexes
for Small Molecule Activation. Acc. Chem. Res..

[13] Solans-Monfort X., Fierro J. L. G., Hermosilla L., Sieiro C., Sodupe M., Mas-Ballesté R. (2011). O-O Bond activation
in H_2_O_2_ and
(CH_3_)_3_C-OOH mediated by [Ni­(cyclam)­(CH_3_CN)_2_]­(ClO_4_)_2_: Different mechanisms
to form the same Ni­(III) product?. Dalton Trans..

[14] Nagataki T., Tachi Y., Itoh S. (2006). Ni^II^(TPA) as an efficient
catalyst for alkane hydroxylation with m-CPBA. Chem. Commun..

[15] Balamurugan M., Mayilmurugan R., Suresh E., Palaniandavar M. (2011). Nickel­(II)
complexes of tripodal 4N ligands as catalysts for alkane oxidation
using *m*-CPBA as oxidant: ligand stereoelectronic
effects on catalysis. Dalton Trans..

[16] Pan H.-R., Wu J., Tsai C.-M., Liao P.-J., Hsu H.-F. (2025). Basicity-Controlled
C-H Bond Activation by a Structurally Characterized Ni­(III)-Hydroxo
Complex. J. Am. Chem. Soc..

[17] Mateos-Calbet A., Bruzzese P. C., Mermigki M. A., Schnegg A., Pantazis D. A., Cornella J. (2025). Rapid Oxygen Atom Transfer
at a Catalysis-Relevant
Ni­(I)-Alkyl Complex with N2O. J. Am. Chem. Soc..

[18] Lyakin O. Y., Talsi E. P., Bryliakov K. P. (2025). Bioinspired Oxidations of C-H Groups
Mediated by Nonheme Complexes of Late Transition Metals Co, Ni, and
Cu. ACS Catal..

[19] Yao S., Bill E., Milsmann C., Wieghardt K., Driess M. (2008). A “Side-on” Superoxonickel
Complex [LNi­(O_2_)] with a Square-Planar Tetracoordinate
Nickel­(II) Center
and Its Conversion into [LNi­(μ-OH)_2_NiL]. Angew. Chem., Int. Ed..

[20] Schröder D., Schwarz H. (1995). C-H and C-C Bond Activation
by Bare Transition-Metal
Oxide Cations in the Gas Phase. Angew. Chem.,
Int. Ed..

[21] Pierpont A. W., Cundari T. R. (2010). Computational Study of Methane C-H Activation by First-Row
Late Transition Metal L_
*n*
_M=E (M:
Fe, Co, Ni) Complexes. Inorg. Chem..

[22] Shiota Y., Yoshizawa K. (2000). Methane-to-Methanol
Conversion by First-Row Transition-Metal
Oxide Ions: ScO^+^, TiO^+^, VO^+^, CrO^+^, MnO^+^, FeO^+^, CoO^+^, NiO^+^, and CuO^+^. J. Am. Chem.
Soc..

[23] Corona T., Pfaff F. F., Acuña-Parés F., Draksharapu A., Whiteoak C. J., Martin-Diaconescu V., Lloret-Fillol J., Browne W. R., Ray K., Company A. (2015). Reactivity of a Nickel­(II)
Bis­(amidate) Complex with meta-Chloroperbenzoic Acid: Formation of
a Potent Oxidizing Species. Chem.—Eur.
J..

[24] Pfaff F. F., Heims F., Kundu S., Mebs S., Ray K. (2012). Spectroscopic
capture and reactivity of S = 1/2 nickel­(III)-oxygen intermediates
in the reaction of a Ni^II^-salt with *m*CPBA. Chem. Commun..

[25] Bok K. H., Lee M. M., You G. R., Ahn H. M., Ryu K. Y., Kim S.-J., Kim Y., Kim C. (2017). Synthesis, Characterization,
and Catalytic Activities of A Nickel­(II) Monoamido-Tetradentate Complex:
Evidence For Ni^III^-Oxo and Ni^IV^-Oxo Species. Chem.—Eur. J..

[26] Karmalkar D. G., Larson V. A., Malik D. D., Lee Y.-M., Seo M. S., Kim J., Vasiliauskas D., Shearer J., Lehnert N., Nam W. (2022). Preparation
and Characterization of a Formally NiIV-Oxo Complex with a Triplet
Ground State and Application in Oxidation Reactions. J. Am. Chem. Soc..

[27] Pfirrmann S., Yao S., Ziemer B., Stösser R., Driess M., Limberg C. (2009). β-Diketiminato
Nickel­(I) Complexes with Very Weak Ligation Allowing for H_2_ and N_2_ Activation. Organometallics.

[28] Pfirrmann S., Limberg C., Herwig C., Stößer R., Ziemer B. (2009). A Dinuclear Nickel­(I)
Dinitrogen Complex and its Reduction
in Single-Electron Steps. Angew. Chem., Int.
Ed..

[29] Kogut E., Wiencko H. L., Zhang L., Cordeau D. E., Warren T. H. (2005). A Terminal
Ni­(III)-Imide with Diverse Reactivity Pathways. J. Am. Chem. Soc..

[30] Bai G., Wei P., Stephan D. W. (2005). Α
β-Diketiminato-Nickel­(II) Synthon for
Nickel­(I) Complexes. Organometallics.

[31] Puiu S. C., Warren T. H. (2003). Three-Coordinate
β-Diketiminato Nickel Nitrosyl
Complexes from Nickel­(I)-Lutidine and Nickel­(II)-Alkyl Precursors. Organometallics.

[32] Holland P. L., Cundari T. R., Perez L. L., Eckert N. A., Lachicotte R. J. (2002). Electronically
Unsaturated Three-Coordinate Chloride and Methyl Complexes of Iron,
Cobalt, and Nickel. J. Am. Chem. Soc..

[33] Chand K., Tsai C.-L., Chen H.-Y., Ching W.-M., Hsu S.-P., Carey J. R., Hsu S. C. N. (2018). Improved
Synthesis of Unsymmetrical
N-Aryl-N′-alkylpyridyl ß-Diketimines Using Molecular Sieves
and their Lithium Complexes. Eur. J. Inorg.
Chem..

[34] Chuang W.-J., Chen H.-Y., Chen W.-T., Chang H.-Y., Chiang M. Y., Chen H.-Y., Hsu S. C. N. (2016). Steric and chelating ring concerns
on the L-lactide polymerization by asymmetric β-diketiminato
zinc complexes. RSC Adv..

[35] Chuang W.-J., Hsu S.-P., Chand K., Yu F.-L., Tsai C.-L., Tseng Y.-H., Lu Y.-H., Kuo J.-Y., Carey J. R., Chen H.-Y., Chen H.-Y., Chiang M. Y., Hsu S. C. N. (2017). Reactivity
Study of Unsymmetrical β-Diketiminato Copper­(I) Complexes: Effect
of the Chelating Ring. Inorg. Chem..

[36] Chand K., Chu Y.-C., Wang T.-W., Kao C.-L., Lin Y.-F., Tsai M.-L., Hsu S. C. N. (2022). Nitric oxide generation study of
unsymmetrical β-diketiminato copper­(II) nitrite complexes. Dalton Trans..

[37] Pahar S., Sharma V., Mahata B., George C. P., Sharma H., Vanka K., Sen S. S. (2022). Tridentate
NacNac Stabilized Tin
and Nickel Complexes: Access to a Monomeric Nickel Hydride and Its
Catalytic Application. Inorg. Chem..

[38] Pahar S., Sharma V., Raj K. V., Sangole M. P., George C. P., Singh K., Vanka K., Gonnade R. G., Sen S. S. (2024). Tridentate
NacNac Tames T-Shaped Nickel­(I) Radical. Chem.—Eur.
J..

[39] Rettenmeier C., Wadepohl H., Gade L. H. (2014). Stereoselective Hydrodehalogenation
via a Radical-Based Mechanism Involving T-Shaped Chiral Nickel­(I)
Pincer Complexes. Chem.—Eur. J..

[40] Rettenmeier C. A., Wadepohl H., Gade L. H. (2016). Electronic structure and reactivity
of nickel­(I) pincer complexes: their aerobic transformation to peroxo
species and site selective C-H oxygenation. Chem. Sci..

[41] Griego L., Woods T. J., Mirica L. M. (2022). A five-coordinate Ni­(I) complex supported
by 1,4,7-triisopropyl-1,4,7-triazacyclononane. Chem. Commun..

[42] Demaison J., Dubrulle A., Boucher D., Burie J., Typke V. (1979). Microwave
spectra, centrifugal distortion constants, and rz structure of acetonitrile
and its isotopic species. J. Mol. Spectrosc..

[43] Michelin R. A., Mozzon M., Bertani R. (1996). Reactions of transition metal-coordinated
nitriles. Coord. Chem. Rev..

[44] Shoshani M. M., Beck R., Wang X., McLaughlin M. J., Johnson S. A. (2018). Synthesis of Surface-Analogue Square-Planar
Tetranuclear
Nickel Hydride Clusters and Bonding to μ_4_-NR, -O
and -BH Ligands. Inorg. Chem..

[45] Stolley R. M., Duong H. A., Thomas D. R., Louie J. (2012). The Discovery of [Ni­(NHC)­RCN]_2_ Species and Their Role as Cycloaddition Catalysts for the
Formation of Pyridines. J. Am. Chem. Soc..

[46] Huang D., Holm R. H. (2010). Reactions of the
Terminal Ni^II^-OH Group
in Substitution and Electrophilic Reactions with Carbon Dioxide and
Other Substrates: Structural Definition of Binding Modes in an Intramolecular
Ni^II^···Fe^II^ Bridged Site. J. Am. Chem. Soc..

[47] Powell-Jia D., Ziller J. W., DiPasquale A. G., Rheingold A. L., Borovik A. S. (2009). A structure and reactivity analysis
of monomeric Ni­(II)-hydroxo
complexes prepared from water. Dalton Trans..

[48] Adhikari D., Mossin S., Basuli F., Dible B. R., Chipara M., Fan H., Huffman J. C., Meyer K., Mindiola D. J. (2008). A Dinuclear Ni­(I)
System Having a Diradical Ni_2_N_2_ Diamond Core
Resting State: Synthetic, Structural, Spectroscopic Elucidation, and
Reductive Bond Splitting Reactions. Inorg. Chem..

[49] Li Y., Fan W., Zhang Z., Xie X., Xiang S., Huang D. (2020). Copper­(II)-hydroxide
facilitated C-C bond formation: the carboxamido pyridine system versus
the methylimino pyridine system. Dalton Trans..

[50] Kieber-Emmons M. T., Schenker R., Yap G. P. A., Brunold T. C., Riordan C. G. (2004). Spectroscopic
Elucidation of a Peroxo Ni2­(μ-O2) Intermediate Derived from
a Nickel­(I) Complex and Dioxygen. Angew. Chem.,
Int. Ed..

[51] Duan P.-C., Manz D.-H., Dechert S., Demeshko S., Meyer F. (2018). Reductive
O_2_ Binding at a Dihydride Complex Leading to Redox Interconvertible
μ-1,2-Peroxo and μ-1,2-Superoxo Dinickel­(II) Intermediates. J. Am. Chem. Soc..

[52] Zhao N., Filatov A. S., Xie J., Hill E. A., Rogachev A. Y., Anderson J. S. (2020). Generation and Reactivity
of a Ni^III2^(μ-1,2-peroxo)
Complex. J. Am. Chem. Soc..

[53] McNeece A. J., Jesse K. A., Xie J., Filatov A. S., Anderson J. S. (2020). Generation
and Oxidative Reactivity of a Ni­(II) Superoxo Complex via Ligand-Based
Redox Non-Innocence. J. Am. Chem. Soc..

[54] Morimoto Y., Takagi Y., Saito T., Ohta T., Ogura T., Tohnai N., Nakano M., Itoh S. (2018). A Bis­(μ-oxido)­dinickel­(III)
Complex with a Triplet Ground State. Angew.
Chem., Int. Ed..

[55] Shiren K., Ogo S., Fujinami S., Hayashi H., Suzuki M., Uehara A., Watanabe Y., Moro-oka Y. (2000). Synthesis, Structures, and Properties
of Bis­(μ-oxo)­nickel­(III) and Bis­(μ-superoxo)­nickel­(II)
Complexes: An Unusual Conversion of a Ni^III2^(*μ*-O)_2_ Core into a Ni^II2^(*μ*-OO)_2_ Core by H_2_O_2_ and Oxygenation
of Ligand. J. Am. Chem. Soc..

[56] Hikichi S., Yoshizawa M., Sasakura Y., Komatsuzaki H., Moro-oka Y., Akita M. (2001). Structural
Characterization and Intramolecular
Aliphatic C-H Oxidation Ability of M^III^(μ-O)_2_M^III^ Complexes of Ni and Co with the Hydrotris­(3,5-dialkyl-4-X-pyrazolyl)­borate
Ligands Tp^Me2,X^ (X = Me, H, Br) and Tp^
*i*Pr2^. Chem.—Eur. J..

[57] Lu T., Chen F. (2012). Multiwfn: A multifunctional
wavefunction analyzer. J. Comput. Chem..

