# Supplementary material for: Identifying a Terminal Nickel–Oxygen Complex Bearing an Unsymmetrical β‑Diketiminate Ligand
Source: JACS Au. 2026 May 8;6(5):2709–14. doi: 10.1021/jacsau.6c00356 (PMC13213496; doi:10.1021/jacsau.6c00356)
Supplement: Supplementary file 1 [file au6c00356_si_001.pdf]

Supporting Information

Identifying a Terminal Nickel-Oxygen Complex Bearing an Unsymmetrical β-Diketiminate Ligand

Si-Hong Chen,[a] Tzu-Hsien Yang,[a] Yu-Lun Chang,[a] Wei-Syuan Lin,[a] Chuan-Hung Chuang,[b] Cheng-Han Yang,[b] Hsing-Yin Chen*,[a], Ming-Li Tsai*,[b], Sodio C. N. Hsu*,[a,b,c]

[a]*Department of Medicinal and Applied Chemistry, Drug Development and Value Creation Research Center, Kaohsiung Medical University, Kaohsiung 80708, Taiwan*
[b]*Department of Chemistry, National Sun Yat-Sen University, Kaohsiung 80424, Taiwan*
[c]*Department of Medical Research, Kaohsiung Medical University Hospital, Kaohsiung 80708, Taiwan*

*E-mail: hychen@kmu.edu.tw (Prof. Hsing-Yin Chen)
mltsai@mail.nsysu.edu.tw (Prof. Ming-Li Tsai)
sodiohsu@kmu.edu.tw (Prof. Sodio C. N. Hsu)

Table of contents

Experimental Section..S5-S11

Tables

Table S1. Selected bond lengths and angles for complexes (**1-5**)………………….S12

Table S2. Crystallographic data for complexes (**1-5**) ……………………………...S13

Table S3. NPA spin densities of complexes **2** and **5**……………………………….S14

Figures

Figure S1. Molecular structure of **1**…………………………………………......…S15

Figure S2A. ^{1}H NMR spectrum of **1** in C_6D_6 (400 MHz, 298 K) ..…………….…..S15

Figure S2B. ^{13}CNMR spectrum of **1** in C_6D_6 (100.58 MHz, 298 K)……………....S16

Figure S3. ESI-MS spectrum of **1** in MeCN……………………………………….....S17

Figure S4. ^{1}H NMR spectrum of **2** in C_6D_6 (400 MHz, 298 K)..………………….....S17

Figure S5. Molecular structure of **4** (Molecule A) ……………..……………….....S18

Figure S6A. ^{1}H NMR spectrum of **4** in C_6D_6 (400 MHz, 298 K)....………....…....S18

Figure S6B. $^{13}C\{H^{1}\}$NMR spectrum of **4** in C_6D_6 (100.58 MHz, 298 K)………....S19

Figure S7. FTIR spectrum (KBr) of **4** (**L**Ni-OH) and **L**Ni-OD…………………....S19

Figure S8. Oxygenation of **2** at -80 °C in toluene by UV titration ……..………..S20

Figure S9. The DFT energy profile of the conversion of **2** to **5**..…………...……..S20

Figure S10. UV–vis spectral changes accompanying oxygenation of **2** in MTHF at -80 ˚C and -110 ˚C…………………………………………………………....S21

Figure S11. Electronic spectrum in toluene of **5** at 193K, 243 K, and 298K.…..….S21

Figure S12. EPR spectra of an *in-situ* generated **5** monitored during the temperature change from 100 K to 280 K in toluene……………..…………..……………….S22

Figure S13. Degradation of complex **5** by EPR spectrum monitoring over time during the temperature at 273 K in toluene.………………………………………….….S22

Figure S14A. EPR spectra of **2** at different concentrations showing linear intensity increase with concentration (inset, $R^2 = 0.99$) in toluene at 100K.……………….…S23

Figure S14B. EPR spectra of **2** and **5** generation by **2** react with O_2 (red line) in toluene at 100K……………………………………………….……………………...…S23

Figure S14C. Comparison of the relative molecular weights of nickel-oxygen

species……………………………………………………….………………….……...….S24
Figure S15A. Oxygenation (N_2O) of **2** at -80 °C in toluene by UV titration………S24
Figure S15B. EPR spectra of **2** (black line) and **5** generation by **2** react with N_2O (red line) in toluene at 100K………..…………………………………..………………S25
Figure S15C. Raman spectra (λ_{ex} = 532 nm) of **2** (black line) and **5** generation by **2** react with N_2O (red line) in MTHF at -80°C………….…………………………....S25
Figure S16A. EPR spectra of **5** generation by **2** react with TMAO in THF solution at 100 K..………………………...……………………………………………….…S26
Figure S16B. UV spectra of **5** generation by **2** react with TMAO in toluene at -80°C…………………………...……………………………………………….…S26
Figure S17. Selected molecular orbitals of the restricted open-shell wavefunction of the symmetrical β-diketiminato nickel-monooxygen complex **5**…....................….S27
Figure S18. ^{31}P NMR spectrum of **5** react with PPh_3(two equivalences) in C_6D_6 (121 MHz, 298 K)……...…………………………………………………………...S28
Figure S19A. EPR Calibration Curve for TEMPO• in Toluene at 298 K…………...S28
Figure S19B. EPR spectrum (298 K) of **5** react with TEMPOH in toluene at 243 K overnight……………………………………………………………………....S29
Figure S20. Electronic spectrum in Toluene after **5** react with TEMPOH at 193 K and warmed to 298K………..…………………………………………………..S29
Figure S21. Electronic spectrum in toluene after **4** react with TEMPO at 298K……S30
Figure S22. Electronic spectrum of **1** in toluene at 298 K…………………………S30
Figure S23. Electronic spectrum of **2** in toluene at 298 K……………………….…S31
Figure S24. EPR spectrum of **2** in toluene at 298 K………………………………..S31
Figure S25. EPR spectrum of **2** in toluene at 193 K……………………………….…S32
Figure S26. Electronic spectrum of **4** in toluene at 298 K……………………….…S32
Figure S27. Electronic spectrum of **5** in toluene at 193 K……………………….....S33
Figure S28. EPR spectrum of **5** in toluene solution at 243 K………………………S33
Figure S29. EPR spectrum of **5** in toluene solution at 108 K……………….……...S34
Figure S30. Electronic spectrum of **1**, **2**, **4**, and **5** in toluene at 193 K…………….…S34
Figure S31. Kinetic UV of **5** react with varying concentrations of TEMPOH in toluene at -30°C…………………………………………………………………S35
Figure S32. X-band EPR spectra of **2** react with 2 equiv PPh_3 and in-situ prepared **5** followed by 2 equiv PPh_3………………………………………………….…..S35

Schemes

Scheme S1. Representation of a nickel-oxygen from the *N*-aryl-*N′*-methylpyridyl β-diketiminato nickel(I) complex with two different oxygen sources and their pathway……………………………………………………………………………..S36

Scheme S2. (a) Synthesis of the *N*-aryl-*N′*-methylpyridyl β-diketiminato nickel complexes. (b) Representations of **5** as a Ni^{III}=O or Ni^{II}–O$^{\bullet}$ species………………..S36

Experimental Section

All manipulations were carried out under a purified dinitrogen atmosphere in a glovebox or using standard Schlenk techniques. Chemical reagents were purchased from Sigma-Aldrich Co. Ltd., Lancaster Chemicals Ltd., or Fluka Ltd. Glassware was dried overnight at 150 °C. All reagents were used without further purification, apart from solvents, which were dried over Na (THF, toluene, hexane, and pentane), CaH_2 (d-benzene and acetonitrile), or extracted from an Innovative Technologies solvent purification system (CH_2Cl_2 and diethyl ether) and then thoroughly degassed before use. LH[1-4] and $[NiCl_2(2,4\text{-lutidine})_2]$[5-6] were synthesized following published procedures. ^1H NMR spectra were acquired on a JEOL JNM ECS 400 MHz. ESI mass spectra were collected using a Waters ZQ 4000 mass spectrometer. UV-vis spectra were recorded on an Agilent 8453 spectrometer adapted with Unisoku USP-203 Cryostat. FTIR spectra were recorded on a Bruker Optics FTIR Alpha OPUS. Elemental analyses were performed using a Heraeus CHN-OS Rapid Elemental Analyzer. EPR spectrum was recorded on an EMXplus-10/12/P/L SYSTEM and EMXnano BENCH-TOP SYSTEM spectrometer.

LNiCl (1). $[NiCl_2(2,4\text{-lutidine})_2]$ (0.495 g, 1.430 mmol) was added to a solution of LH (0.500 g, 1.430 mmol) in 15 mL THF, and the mixture was stirred for 1 h. A solution of KO^tBu (0.160 g, 1.430 mmol) in 10 mL THF was added dropwise to the mixture, and the mixture was stirred for another 3 h. The solvent was removed under vacuum, and the residue was extracted with 30 mL of CH_2Cl_2. The solution was filtered through Celite, and the filtrate was evaporated to dryness under vacuum. The residual solid was washed with hexane and dried under vacuum to yield a brown solid of **1** (0.500, 1.129 mmol, 79%). A single crystal of **1** was obtained by cooling the saturated THF solution to -35°C for 2 days. Anal. Calcd for $C_{23}H_{30}N_3NiCl$: C, 62.41; H, 8.83; N, 9.49. Found: C, 62.44; H, 8.81; N, 9.52. Found: C, 62.44; H, 8.81; N, 9.52. ^1H NMR (C_6D_6, 400 MHz, 298 K, δ): 9.24 (d, 1H, J = 6.0 Hz, Py1), 7.26-7.17 (m, 3H, Ar-*H*), 6.51 (t, 1H, Py2), 6.10 (t, 1H, Py3), 5.91 (d, 1H, J = 7.8 Hz, Py4), 4.92 (s, 1H, backbone-C*H*), 4.36 (septet, 2H, ArC*H*$(CH_3)_2$), 3.65 (s, 2H, CH_2Py), 1.99 (d, 6H, J =

6.8 Hz, ArCH(CH_3)$_2$), 1.60 (s, 3H, backbone-CH_3), 1.54 (s, 3H, backbone-CH_3), 1.32 (d, 6H, J = 6.9 Hz, ArCH(CH_3)$_2$). $^{13}C\{^1H\}$ NMR (C_6D_6, 100.58 MHz, 298 K, δ): 162.14, 160.68, 157.36, 151.67, 149.75, 141.77, 135.94, 125.42, 123.06, 121.06, 117.54, 98.61, 60.82, 28.91, 24.76, 24.56, 24.08, 20.90; UV-vis (toluene, 298 K, ε in $M^{-1}cm^{-1}$): 359 (6330), 386 (6775), 504 (142), 657 (71).

LNi (2). Potassium graphite (KC_8) (0.080 g, 0.590 mmol) was added to a solution of **1** (0.200 g, 0.454 mmol) in 20 mL THF. The solution turned from brown to deep red immediately, and the mixture was stirred for 2 h. The solvent was removed under vacuum, and the residue was extracted with 100 mL of pentane. The solution was filtered through a 0.22 μm syringe filter, and the filtrate was evaporated to dryness under vacuum to yield a deep red solid of **2** (0.143 g, 0.352 mmol, 96.2%). A single crystal of **3** was obtained by cooling the saturated THF solution of **2** to -35°C for 3 days. ^1H NMR (C_6D_6, 400 MHz, 298 K, δ): 30.02, 19.24, 16.47 (sh), 11.54, 4.10, 1.64, 1.26, -3.61, -11.79, -22.72. UV-vis (toluene, 298 K, ε in $M^{-1}cm^{-1}$): 305 (sh, 8694), 392 (3306), 411 (3627), 555 (2312). EPR (toluene, 100 K, Figure 1): g_1 = 2.288, g_2 = 2.134, g_3 = 2.035.

LNiOH (4). Potassium hydroxide (0.031 g, 0.548 mmol) was added to a solution of **1** (0.200 g, 0.454 mmol) in 30 mL THF. The solution gradually turned from brown to olive green, and the mixture was stirred for 12 h. The solvent was removed under vacuum, and the residue was extracted with 80 mL of diethyl ether. The solution was filtered through a 0.22 μm syringe filter, and the filtrate was evaporated to dryness under vacuum to yield a green solid of **4** (0.156 g, 0.369 mmol, 82%). Single crystals of **4** were obtained by cooling the saturated diethyl ether solution to -35°C for 2 days. Anal. Calcd for $C_{23}H_{31}N_3NiO$: C, 65.12; H, 7.37; N, 9.91. Found: C, 65.14; H, 7.41; N, 9.82. ^1H NMR (C_6D_6, 400 MHz, 298 K, δ): 9.27 (d, 1H, J = 4.8 Hz, Py1), 7.18 (s, 3H, Ar-*H*), 6.67 (t, 1H, Py2), 6.38 (t, 1H, Py3), 6.05 (d, 1H, J = 7.8 Hz, Py4), 4.99 (s, 1H, backbone-C*H*), 4.30 (septet, 2H, ArC*H*(CH$_3$)$_2$), 3.64 (s, 2H, CH_2Py), 1.92 (d, 6H, J = 6.8 Hz, ArCH(CH_3)$_2$), 1.65 (s, 3H, backbone-CH_3), 1.53 (s, 3H, backbone-CH_3), 1.30 (d, 6H, J = 7.0 Hz, ArCH(CH_3)$_2$), -4.73 (s, 1H, O*H*). $^{13}C\{^1H\}$ NMR (C_6D_6,

100.58 MHz, 298 K, δ): 161.98, 159.45, 158.19, 148.06, 145.18, 143.21, 135.29, 125.83, 123.63, 120.60, 117.43, 98.01, 59.08, 28.44, 25.11, 23.91, 23.48, 21.11; UV-vis (toluene, 298 K, ε in $M^{-1}cm^{-1}$): 329 (4556), 342 (4195), 400 (3635), 430 (sh, 2199), 579 (98). FTIR (KBr): ν(O−H) = 3637 cm^{-1}

LNiO (5). A toluene solution of **2** (0.200 g, 0.491 mmol) was concentrated under reduced pressure at ambient temperature until near saturation. Fresh dry toluene (2–3 drops) was added to slightly undersaturate the solution and prevent overly rapid solidification upon cooling. The solution was then placed in a −40%˚C cooling bath, and 0.5%equiv of dry oxygen (6 mL, 0.245 mmol) was slowly added, resulting in a color change from rose-red to orange-red. The mixture was allowed to stand for 15%min, after which slow cooling was initiated by decreasing the temperature by 5%˚C every 30%min. The solution was subsequently cooled to −80%˚C and left overnight. On the following day, red crystals were observed adhering to the walls of the flask as single crystals of **5** (0.054 g, 0.128 mmol, 26%). UV-vis (toluene, 193 K, ε in $M^{-1}cm^{-1}$): 330 (4451), 346 (4542), 370 (4521), 430 (sh, 1769), 505 (713.4). EPR (toluene, 100 K, Figure 6): g_1 = 2.110, g_2 = 2.008, g_3 = 2.004. Owing to the highly thermal sensitivity of **5**, the elemental analysis could not be performed.

Spectrophotometric O_2 Titrations

The O_2-saturated toluene solution (8 mM) was prepared by bubbling the dry O_2 gas through nitrogen-saturated toluene in a 25 mL round bottom flask at 25 °C for 30 min.[7] The experiment procedure at −80 °C mentioned here follows the details as reported in the literature.[8-10] A 4.0 mL toluene solution of complex **2** (0.12 mM) was placed in a UV–vis cuvette and cooled to −80 °C. During the titration, aliquots of O_2-saturated toluene solution (3.0 μL per addition, corresponding to 0.1 equiv of O_2) were sequentially introduced into the cuvette using a syringe. After each addition, the solution was stirred for 2 min to ensure complete reaction, and the titration was continued until a total of 1.0 equiv of O_2 had been added. The Ni/O_2 stoichiometry was determined to be 2:1 by monitoring the absorbance changes at 347 and 523 nm in the UV–vis spectra.

Spectrophotometric N_2O Titrations

An N_2O-saturated toluene solution was prepared by bubbling dry N_2O through degassed toluene in a 25 mL round-bottom flask at 25 °C for 30 min. Under these conditions, the concentration of dissolved N_2O was taken as 12 mM.[11] A 4.0 mL toluene solution of complex **2** (0.12 mM) was placed in a UV–vis cuvette and cooled to −80 °C. During the titration, aliquots of N_2O-saturated toluene (4.0 μL per addition, corresponding to 0.1 equiv relative to **2**) were sequentially introduced into the cuvette using a microsyringe. After each addition, the solution was stirred for 5 min to ensure complete reaction, and the titration was continued until a total of 1.0 equiv of N_2O had been added. The Ni/N_2O stoichiometry was determined to be 1:1 by monitoring the absorbance changes at 347 and 523 nm in the UV–vis spectra.

Oxygenation of 2 by Trimethylamine N-oxide

Trimethylamine N-oxide (TMAO) was purified by three successive sublimations before use. A 4.0 mL toluene solution of **2** (0.12 mM, 0.00048 mmol) was placed in a UV–vis cuvette and cooled to −80 °C. A suspension of TMAO (0.180 mg, 0.00240 mmol, 5 equiv) in toluene (0.1 mL) was then added by syringe. The resulting mixture was stirred at −80 °C, and the reaction was monitored by UV–vis spectroscopy. Changes in the absorption bands were recorded over time to follow the formation of the oxygenated nickel species.

Oxidation of PPh_3

A solution of **2** (0.005 g, 0.0123 mmol) in C_6D_6 (0.5 mL) was placed in a cooling bath at −80 °C. Dry O_2 gas (150 μL, 0.00615 mmol, 0.5 equiv; measured at 1 atm and 298 K) was introduced very slowly via a gas-tight syringe, with the needle positioned at the bottom of the NMR tube.[12] The gas was delivered as a gentle stream, allowing gradual dissolution while minimizing bubble formation and avoiding agitation of the solution, thereby ensuring controlled delivery of O_2. The reaction mixture was stirred for 15 min, during which the color changed from rose-red to orange-red. Subsequently, a solution of PPh_3 (0.00645 g, 0.0246 mmol, 2 equiv) in C_6D_6 (0.1 mL), pre-cooled to −80 °C, was added by syringe. The reaction mixture was then gradually warmed to −30 °C and maintained at this temperature overnight.The resulting solution was

analyzed by ^{31}P NMR spectroscopy. The amount of $OPPh_3$ formed was quantified by integration relative to the residual PPh_3 resonance. Integration of the ^{31}P NMR signals indicated formation of 0.3 equiv of $OPPh_3$.

Hydrogen Atom Abstraction (HAA) of TEMPOH

A solution of **2** (0.0005 g, 0.00123 mmol) in toluene (0.5 mL) was transferred to an EPR tube and cooled to −80 ˚C. Dry O_2 gas (15.0 μL, 0.000615 mmol, 0.5 equiv; measured at 1 atm and 298 K) was introduced very slowly via a gas-tight syringe, with the needle positioned at the bottom of the tube. The gas was delivered as a gentle stream, allowing gradual dissolution while minimizing bubble formation and avoiding agitation of the solution, thereby ensuring controlled generation of **5**. The reaction mixture was allowed to stand for 15 min, during which the color changed from rose-red to orange-red. Subsequently, a solution of TEMPOH (0.000192 g, 0.00123 mmol, 1 equiv) in toluene (0.1 mL), pre-cooled to −80 ˚C, was added by syringe. After addition, the reaction mixture was maintained at −30 ˚C overnight. The following day, the sample was analyzed by EPR spectroscopy, and the concentration of the radical species was determined by spin quantification. Integration of the EPR signal indicated formation of TEMPO• in 76.8% yield.

X-ray crystal structure determinations. All single X-ray diffraction data were accumulated using Rigaku Oxford Diffraction single crystal X-ray diffractometers with Mo K_α radiation (λ = 0.71073 Å). The data collection was executed using the CrysAlisPro 1.171.41.56a program. Cell refinement and data reduction were made with the CrysAlisPro 1.171.41.56a program. The structure was determined using the Olex2/ ShelXL program, refined using full-matrix least squares. All non-hydrogen atoms were refined anisotropically, whereas hydrogen atoms were placed at calculated positions and included in the final stage of refinement with fixed parameters.

EPR Measurements. The low-temperature and variable-temperature EPR spectra of the Ni complexes **2** and **5** dissolved in toluene solution were measured at 100 K via a Bruker EMX plus/EMX-nano spectrometer. In a typical experiment, 0.4 mL of a 2.2 mM solution of the **2** and **5** in toluene was introduced into a gas-tight EPR tube (Willmad 707-LPV-200M) under anaerobic conditions. The EPR tube was placed in a

liquid nitrogen bath and waited for 5 min to equilibrate the temperature prior to measurements. The experimental parameters: microwave frequency = 9.628GHz, microwave power = 0.32 mW, modulation amplitude = 0.5 mT, modulation frequency = 100 kHz, received gain = 40, time constant = 1.28 ms for **2**; microwave frequency = 9.628 GHz, microwave power = 0.32 mW, modulation amplitude = 0.5 mT, modulation frequency = 100 kHz, received gain = 40, time constant = 1.28 ms for *in-situ* $^{16}O_2$-/generated **2**. Spin Hamiltonian parameters of **2** and **5** were determined by fitting the spectrum using Aniso-SpinFit program installed in the instrument.

Raman Measurements. Low-temperature liquid-phase Raman spectra of in-situ $^{16}O_2$- and $^{18}O_2$-generated **5** were recorded at −80 °C in MTHF solution. In a typical experiment, complex **2** was dissolved in freshly distilled MTHF and transferred into a gas-tight Raman tube under anaerobic conditions. The solution was cooled to −80 °C using a cryogenic bath, and $^{16}O_2$ or $^{18}O_2$ gas was introduced via a stainless-steel needle to generate the corresponding Ni–O intermediates. After allowing the reaction to proceed for several minutes to reach equilibrium, the Raman spectrum was measured directly in the liquid phase. Raman spectra were recorded on a GMD532A Raman microscope equipped with a 10× objective lens, a 532 nm continuous-wave excitation laser (100 mW), and a CCD detector cooled to 5 °C. The spectrometer provides a Raman shift range of 150–1950 cm^{-1} with a pixel-to-pixel resolution of 1.3 cm^{-1}. Each spectrum was collected with an acquisition time of 5 s and averaged over 20 scans to improve the signal-to-noise ratio. All spectra were baseline-corrected, and isotopic shifts were analyzed by comparing the $^{16}O_2$- and $^{18}O_2$-derived samples under identical conditions.

Computational methods. The density functional theory calculations were accomplished by the Gaussian 16 program.[13] Geometry optimizations and vibrational frequency calculations were carried out at TPSSTPSS/Def2-SVP level in toluene treated by SMD solvation model.[14] Single point energy calculations at TPSSh/Def2-TZVP level were performed to provide more accurate energies. The thermal correction was made at experimental conditions of -80 °C (193 K), and the concentration conditions used in the calculations were selected based on the 2:1 Ni/O_2 ratio observed in the spectrophotometric titration experiment. The setting of ultrafine grids was adopted for numerical integrations. The atomic charge and spin density were provided by the natural population analysis. The bond order was analyzed by using the Multiwfn program.[15]

References

1. Chand, K.; Tsai, C.-L.; Chen, H.-Y.; Ching, W.-M.; Hsu, S.-P.; Carey, J. R.; Hsu, S. C. N., Improved Synthesis of Unsymmetrical N-Aryl-N´-alkylpyridyl ß-Diketimines Using Molecular Sieves and their Lithium Complexes. *Eur. J. Inorg. Chem.* **2018,** *2018* (9), 1093-1098.
2. Chuang, W.-J.; Chen, H.-Y.; Chen, W.-T.; Chang, H.-Y.; Chiang, M. Y.; Chen, H.-Y.; Hsu, S. C. N., Steric and chelating ring concerns on the L-lactide polymerization by asymmetric β-diketiminato zinc complexes. *RSC Adv.* **2016,** *6* (43), 36705-36714.
3. Chuang, W.-J.; Hsu, S.-P.; Chand, K.; Yu, F.-L.; Tsai, C.-L.; Tseng, Y.-H.; Lu, Y.-H.; Kuo, J.-Y.; Carey, J. R.; Chen, H.-Y.; Chen, H.-Y.; Chiang, M. Y.; Hsu, S. C. N., Reactivity Study of Unsymmetrical β-Diketiminato Copper(I) Complexes: Effect of the Chelating Ring. *Inorg. Chem.* **2017,** *56* (5), 2722-2735.
4. Huang, Y.-C.; Chen, H.-Y.; Chang, Y.-L.; Vasanthakumar, P.; Chen, S.-Y.; Kao, C.-L.; Wu, C. H.-Y.; Hsu, S. C. N., Synthesis of triisocyanomesitylene β-diketiminato copper(I) complexes and evaluation of isocyanide π-back bonding. *Polyhedron* **2020,** *192*, 114828.
5. Wiencko, H. L.; Kogut, E.; Warren, T. H., Neutral β-diketiminato nickel(II) monoalkyl complexes. *Inorg. Chim. Acta* **2003,** *345*, 199-208.
6. Buffagni, S.; Vallarino, L. M.; Quagliano, J. V., Coordination Compounds of Nickel(II) with Substituted Pyridines. Square-Planar, Tetrahedral, and Octahedral Compounds of 2,3-, 2,4-, and 2,5-Dimethylpyridine. *Inorg. Chem.* **1964,** *3* (4), 480-486.
7. Li, A.; Tang, S.; Tan, P.; Liu, C.; Liang, B., Measurement and Prediction of Oxygen Solubility in Toluene at Temperatures from 298.45 K to 393.15 K and Pressures up to 1.0 MPa. *Journal of Chemical & Engineering Data* **2007,** *52* (6), 2339-2344.
8. Chand, K.; Meitei, N. J.; Chang, Y.-L.; Tsai, C.-L.; Chen, H.-Y.; Hsu, S. C. N., Ligand Degradation Study of Unsymmetrical β-Diketiminato Copper Dioxygen Adducts: The Length Chelating Arm Effect. *ACS Omega* **2023,** *8* (23), 21096-21106.
9. Gupta, A. K.; Tolman, W. B., Cu(I)/O2 Chemistry Using a β-Diketiminate Supporting Ligand Derived from N,N-Dimethylhydrazine: A [Cu3O2]3+ Complex with Novel Reactivity. *Inorganic chemistry* **2012,** *51* (3), 1881-1888.
10. Hong, S.; Hill, L. M. R.; Gupta, A. K.; Naab, B. D.; Gilroy, J. B.; Hicks, R. G.; Cramer, C. J.; Tolman, W. B., Effects of Electron-Deficient β-Diketiminate and Formazan Supporting Ligands on Copper(I)-Mediated Dioxygen Activation. *Inorganic chemistry* **2009,** *48* (10), 4514-4523.
11. Chang, C. J.; Chen, C.-Y.; Lin, H.-C., Solubilities of Carbon Dioxide and Nitrous Oxide in Cyclohexanone, Toluene, and N,N-Dimethylformamide at Elevated Pressures. *Journal of Chemical & Engineering Data* **1995,** *40* (4), 850-855.
12. Fischer, K.; Noll, O.; Gmehling, J., Experimental Determination of the Oxygen Solubility in Benzene. *Journal of Chemical & Engineering Data* **2001,** *46* (6), 1504-1505.
13. Frisch, M. J.; Trucks, G. W.; Schlegel, H. B.; Scuseria, G. E.; Robb, M. A.; Cheeseman, J. R.; Scalmani, G.; Barone, V.; Petersson, G. A.; Nakatsuji, H.; Li, X.; Caricato, M.; Marenich, A. V.; Bloino, J.; Janesko, B. G.; Gomperts, R.; Mennucci, B.; Hratchian, H. P.; Ortiz, J. V.; Izmaylov, A. F.; Sonnenberg, J. L.; Williams-Young, D.; Ding, F.; Lipparini, F.; Egidi, F.; Goings, J.; Peng, B.; Petrone,

A.; Henderson, T.; Ranasinghe, D.; Zakrzewski, V. G.; Gao, J.; Rega, N.; Zheng, G.; Liang, W.; Hada, M.; Ehara, M.; Toyota, K.; Fukuda, R.; Hasegawa, J.; Ishida, M.; Nakajima, T.; Honda, Y.; Kitao, O.; Nakai, H.; Vreven, T.; Throssell, K.; Montgomery, J. A., Jr.; Peralta, J. E.; Ogliaro, F.; Bearpark, M. J.; Heyd, J. J.; Brothers, E. N.; Kudin, K. N.; Staroverov, V. N.; Keith, T. A.; Kobayashi, R.; Normand, J.; Raghavachari, K.; Rendell, A. P.; Burant, J. C.; Iyengar, S. S.; Tomasi, J.; Cossi, M.; Millam, J. M.; Klene, M.; Adamo, C.; Cammi, R.; Ochterski, J. W.; Martin, R. L.; Morokuma, K.; Farkas, O.; Foresman, J. B.; Fox, D. J. *Gaussian 16*. Revision C.02; Gaussian, Inc.: Wallingford, CT, 2016.

14. Marenich, A. V. C., C. J.; Truhlar, D. G., Universal Solvation Model Based on Solute Electron Density and on a Continuum Model of the Solvent Defined by the Bulk Dielectric Constant and Atomic Surface Tensions. *J. Phys. Chem. B* **2009,** *113*, 6378−6396.

15. Lu, T.; Chen, F., Multiwfn: A multifunctional wavefunction analyzer. *J. Comput. Chem.* **2012,** *33* (5), 580-592.

Table S1. Selected bond lengths (Å) and angles (deg) for complexes **L**NiCl(**1**), **L**Ni(**2**), (**L**Ni)$_2$μ-κ^1:η^2-NCMe(**3**), **L**NiOH(**4**), and **L**NiO (**5**).

	**L**NiCl(**1**)		**L**Ni (**2**)	(**L**Ni)$_2$μ-κ^1:η^2-NCMe(**3**)	**L**NiOH(**4**)		**L**NiO(**5**)
	Molecule A	Molecule B			Molecule A	Molecule B	
Ni-O					1.854(2)	1.9066(19)	1.750(4)
Ni-Cl	2.2294(10)	2.2090(9)					
Ni-N$_{acetonitrile(\kappa 1)}$				1.879(4)			
Ni-NC$_{acetonitrile(\eta 2)}$				1.917(4)			
Ni-N$_{amide(aryl)}$	1.879(3)	1.883(3)	1.863(3)	1.889(5), 1.894(5)	1.883(2)	1.882(2)	1.866(3)
Ni-N$_{amide(alkyl)}$	1.848(3)	1.840(3)	1.898(3)	1.892(4), 1.914(5)	1.882(2)	1.8744(19)	1.886(3)
Ni-N$_{py}$	1.907(3)	1.919(3)	1.897(3)	1.933(5)	1.909(2)	1.911(2)	1.891(3)
C-C$_{(NCCCN\ backone)}$	1.355(5)<br>1.341(5)	1.352(5)<br>1.342(5)	1.395(5)<br>1.401(5)	1.384(8), 1.405(8)<br>1.390(8), 1.404(8)	1.349(4)<br>1.405(4)	1.381(3)<br>1.395(4)	1.388(5)<br>1.392(5)
C-N$_{(NCCCN\ backone)}$	1.388(5)<br>1.369(5)	1.388(4)<br>1.369(5)	1.347(4)<br>1.336(4)	1.359(7), 1.336(7)<br>1.310(7), 1.315(7)	1.348(3)<br>1.305(3)	1.342(3)<br>1.322(3)	1.348(5)<br>1.329(5)
C-C$_{acetonitrile(\eta 2)}$				1.508(8)			
N$_{amide}$-Ni-N$_{amide}$	89.63(12)	89.75(12)	98.98(12)	94.2(2), 96.83(19)	95.43(9)	94.85(9)	96.16(12)
N$_{amide(aryl)}$-Ni-N$_{py}$	175.13(13)	176.72(12)	173.64(12)	170.30(19), 149.8(2)	175.61(9)	171.42(9)	178.36(12)
N$_{amide(alkyl)}$-Ni-N$_{py}$	87.92(12)	87.94(12)	85.73(13)	83.97(19)	84.56(10)	84.38(9)	84.71(13)
∠NCCCN, aryl[a]	79.79	79.25		86.74, 80.46	81.77	77.20	83.99

[a] ∠angle between the NCCCN backbone plane and *N*-aryl ring.

Table S2. Crystallographic data for complexes **L**NiCl(**1**), **L**Ni(**2**), (**L**Ni)$_2$μ-κ^1:η^2-NCMe(**3**), **L**NiOH(**4**), and **L**NiO (**5**).

	**L**NiCl	**L**Ni	(**L**Ni)$_2$μ-κ^1:η^2-NCMe · (NCMe)$_3$	**L**NiOH[a]	**L**NiO
empirical formula	$C_{46}H_{60}N_6Ni_2Cl_2$	$C_{46}H_{60}N_6Ni_2$	$C_{54}H_{72}N_{10}Ni_2$	$C_{46}H_{62}N_6Ni_2O_2$	$C_{23}H_{30}N_3NiO$
formula weight	885.32	814.42	978.60	848.43	413.61
T (K)	113(2)	130(2)	113(2)	113(2)	113(2)
cystal size (mm^3)	0.3 × 0.15 × 0.15	0.2 × 0.2× 0.1	0.15 × 0.15 × 0.1	0.3 × 0.1 × 0.1	0.13 × 0.1 × 0.1
crystal system	orthorhombic	triclinic	triclinic	monoclinic	monoclinic
space group	Pca2$_1$	P-1	P-1	P2$_1$/n	P2$_1$/n
a (Å)	13.8777(2)	8.3184(2)	11.8438(4)	12.4997(2)	11.6071(3)
b (Å)	14.9143(2)	15.9660(5)	15.4035(7)	19.1342(3)	13.2393(3)
c (Å)	21.0209(2)	18.0353(4)	15.7113(4)	18.6188(3)	14.0699(3)
α (deg)	90	116.060(3)	107.286(3)	90	90
β (deg)	90	90.625(2)	101.008(3)	103.9110(10)	94.354(2)
γ (deg)	90	96.061(2)	102.388(3)	90	90
V (Å^3)	4350.83(10)	21355.19(11)	2571.51(17)	4322.48(12)	2155.88(9)
Z	4	2	5	4	4
D_{calcd} (g cm^{-3})	1.352	1.267	1.264	1.304	1.304
μ (mm^{-1})	1.028	0.920	0.778	0.915	0.917
reflns mcasd/indep	104887/7643	868.0	46183/8927	47159/7599	4723/4482
data/rcstraints/params	7643/1/518	8881/0/499	8927/1860/611	7599/1420/525	3509/685/259
GOF	1.067	1.052	1.041	1.036	1.065
R_{int}	0.0411	0.0757	0.1313	0.0338	0.0615
R_1 [$I > 2\sigma$] (all data)	0.0291(0.0308)	0.00778(0.1493)	0.0839(0.1181)	0.0385(0.0460)	0.0638(3509)
R_w [$I > 2\sigma$] (all data)	0.0790(0.0800)	0.1195(0.1626)	0.232(0.2477)	0.0968(0.1003)	0.2048(4482)
max. peak/hole (e$^-$/ Å^3)	1.22/-0.32	0.98/-0.53	1.98/-0.78	1.04/-0.65	0.46/-1.91

[a] For Alert B on check cif report of **4**, there is no suitable acceptor close to the terminal OH hydrogen atoms. Explanation: The presence of the terminal OH groups were unambiguously evidenced by H NMR and Solid-state FTIR.

Table S3. NPA spin densities of complexes **2** and **5**

	LNi (**2**)	LNiO (**5**)
O	-	0.670
Ni	0.836	0.277
N1	0.035	0.027
N2	0.112	0.020
N3	0.035	0.001

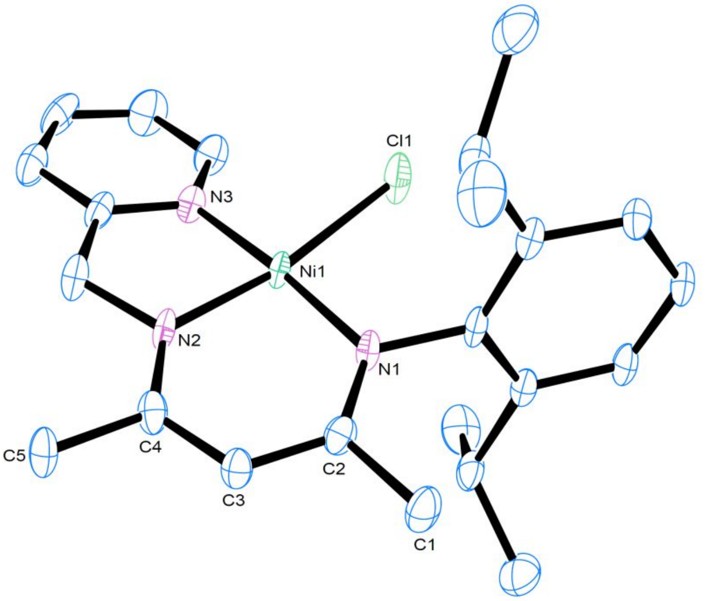

Figure S1. Molecular structure of **1**. Thermal ellipsoids are drawn at a 50% probability level. Hydrogen atoms are omitted for clarity. Selected bond lengths (Å) and angles (deg): Ni(1)-N(1) = 1.879 (3); Ni(1)-N(2) = 1.848(3); Ni(1)-N(3) = 1.907(3); Ni(1)-Cl(1) = 2.2294 (10); N(1)-Ni(1)-N(3) = 175.13(13); N(2)-Ni(1)-Cl(1) = 170.49(9).

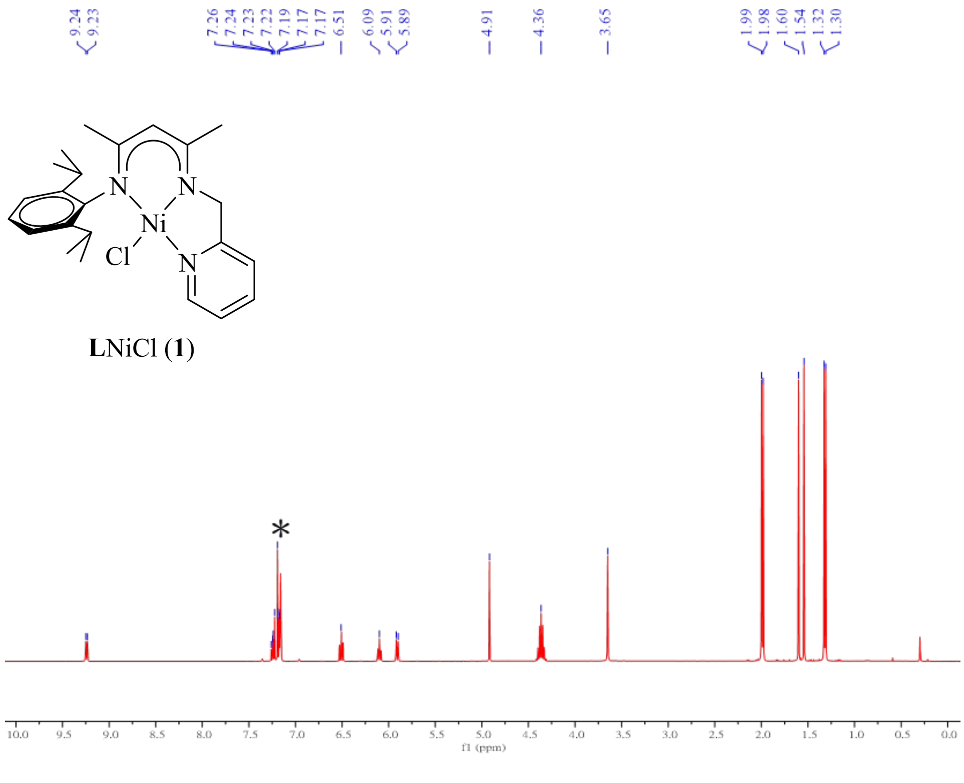

Figure S2A. ^{1}H NMR spectrum of **1** in C_6D_6 (400 MHz, 298 K). Solvent residual peaks are marked with an asterisk (*).

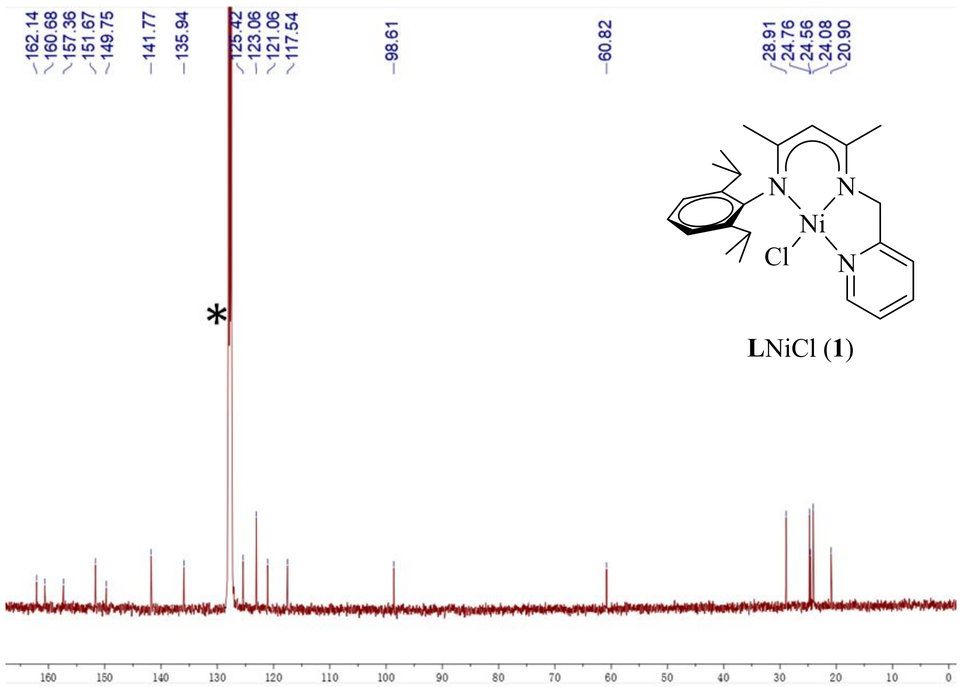

Figure S2B. $^{13}C\{H^1\}$NMR spectrum of **1** in C_6D_6 (100.58 MHz, 298 K). Solvent residual peaks are marked with an asterisk (*).

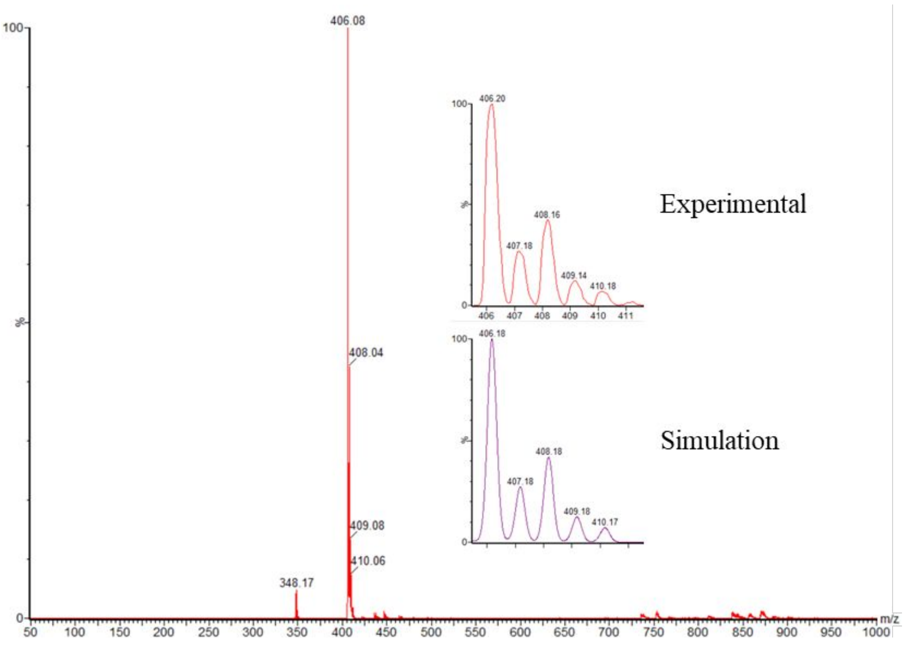

Figure S3. ESI-MS spectrum of **1** in MeCN. The inset shows the experimental (top) and simulated (bottom) isotope pattern for the major peak around *m/z* = 406.18 $[L+M]^+$.

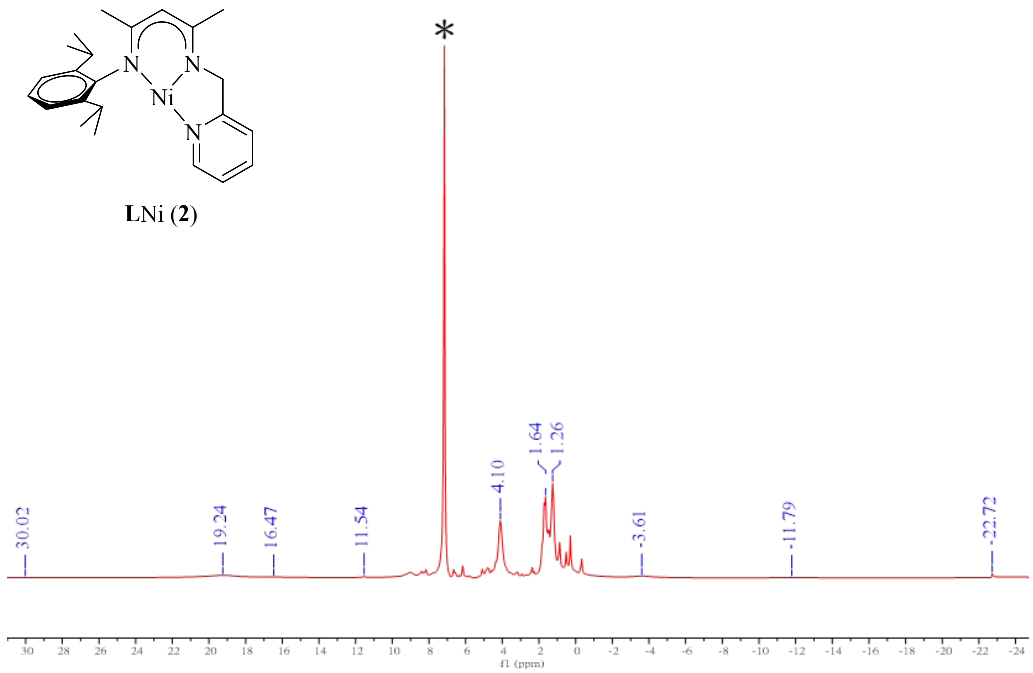

Figure S4. ^{1}H NMR spectrum of **2** in C_6D_6 (400 MHz, 298 K). Solvent residual peaks are marked with an asterisk (*).

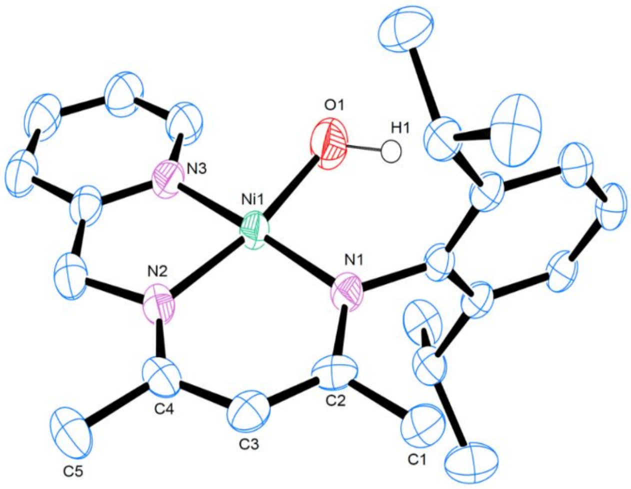

Figure S5. Molecular structure of **4** (Molecule A). Thermal ellipsoids are drawn at a 50% probability level. Hydrogen atoms are omitted for clarity. Selected bond lengths (Å) and angles (deg): Ni(1)-N(1) = 1.883 (2); Ni(1)-N(2) = 1.882(2); Ni(1)-N(3) = 1.909(2); Ni(1)-O(1) = 1.854 (2); N(1)-Ni(1)-N(3) = 175.61 (9); N(2)-Ni(1)-O(1) = 169.63(9).

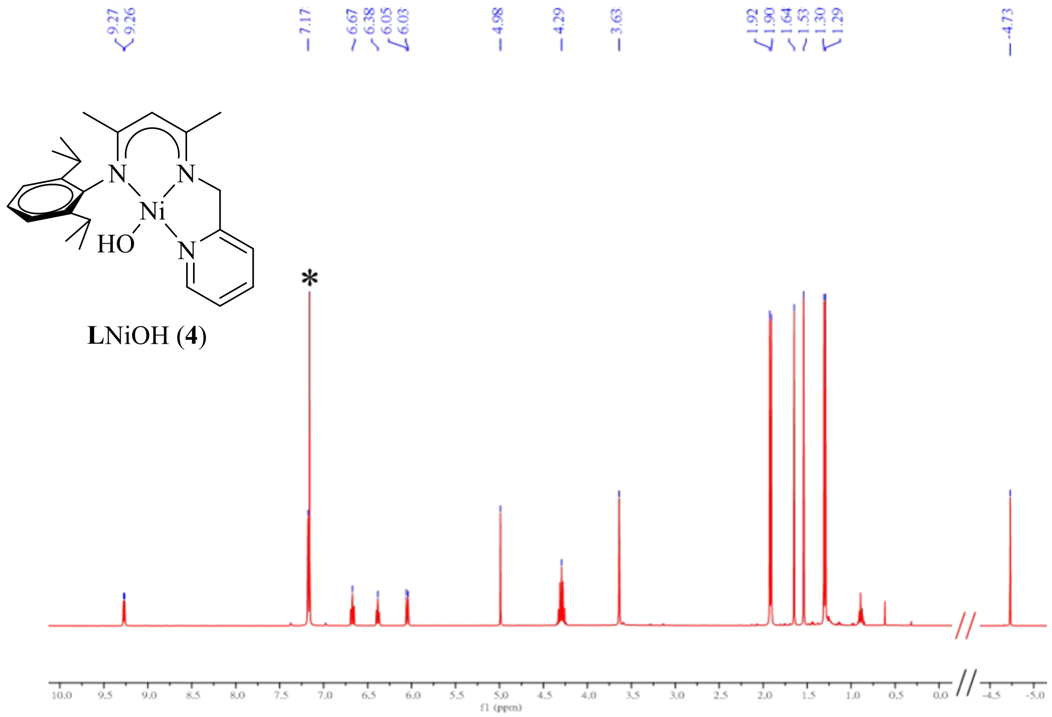

Figure S6A. ^{1}H NMR spectrum of **4** in C_6D_6 (400 MHz, 298 K). Solvent residual peaks are marked with an asterisk (*).

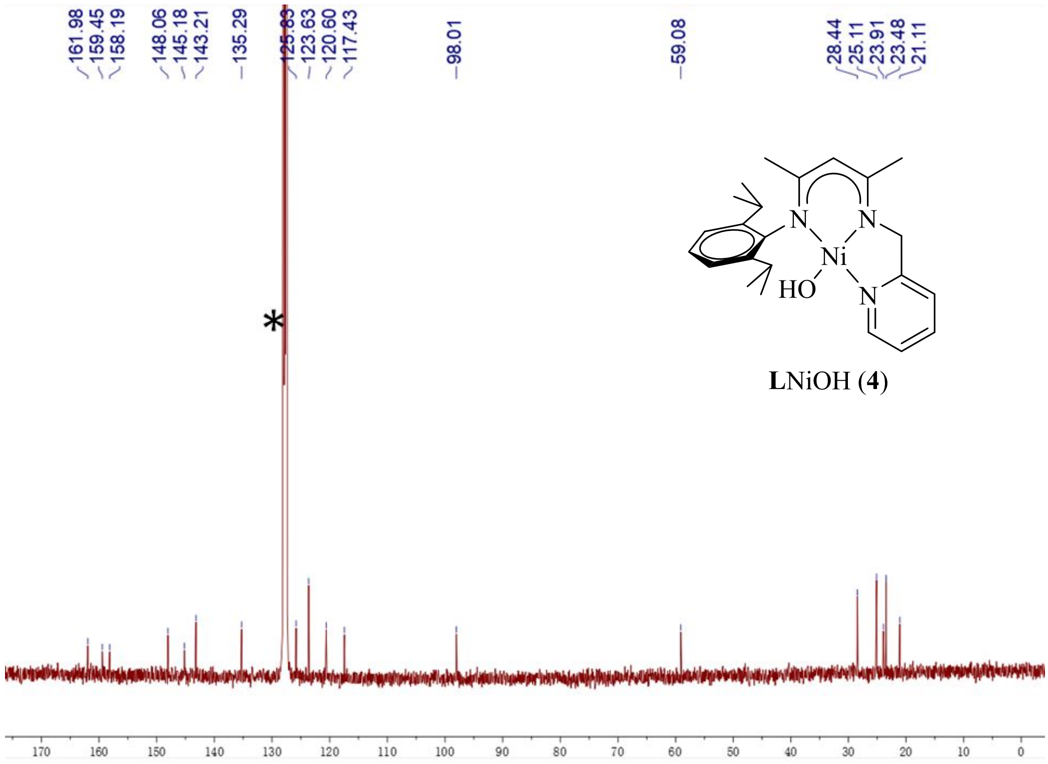

Figure S6B. $^{13}C\{^{1}H\}$ NMR spectrum of **4** in C_6D_6 (100.58 MHz, 298 K). Solvent residual peaks are marked with an asterisk (*).

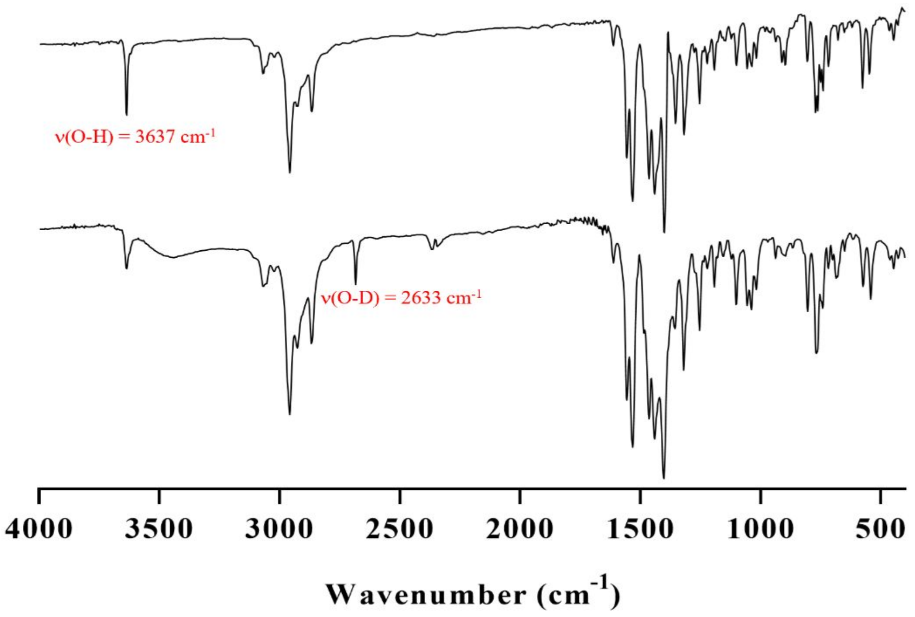

Figure S7. FTIR spectrum (KBr) of **4** (**L**Ni-OH) (top) and its deuteration product **L**Ni-OD (bottom)

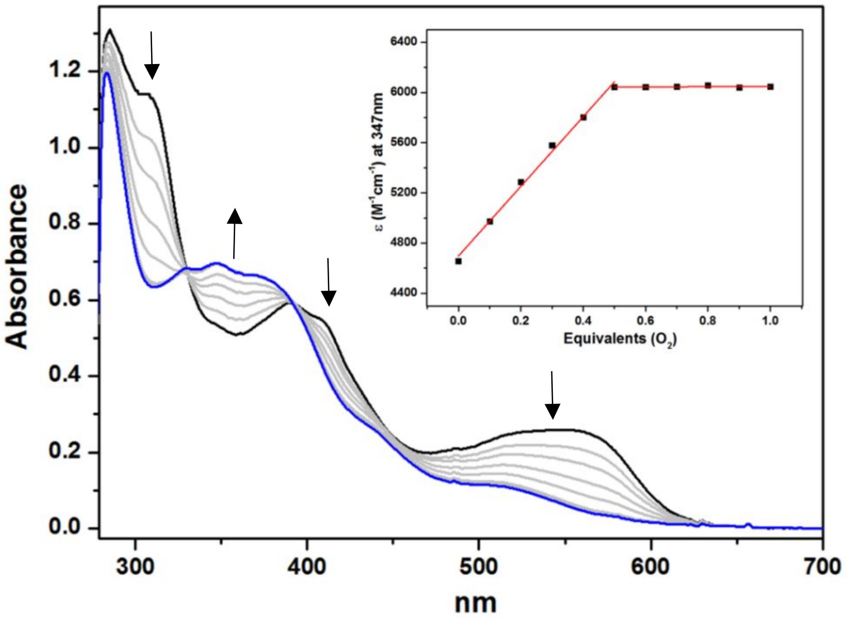

Figure S8. UV-vis changes accompanying oxygenation of **2** at -80 °C in toluene, using starting concentrations of 0.12 mM. The 0.1 equivalent of oxygen was added into **2** per minute until 1.0 equivalent. The initial and final spectra are represented in black and blue, respectively. The insets show the results of spectrophotometric titrations of complex **2** with O_2 at 347 nm, plotted as linear fits at a 2:1 Ni/O_2 ratio.

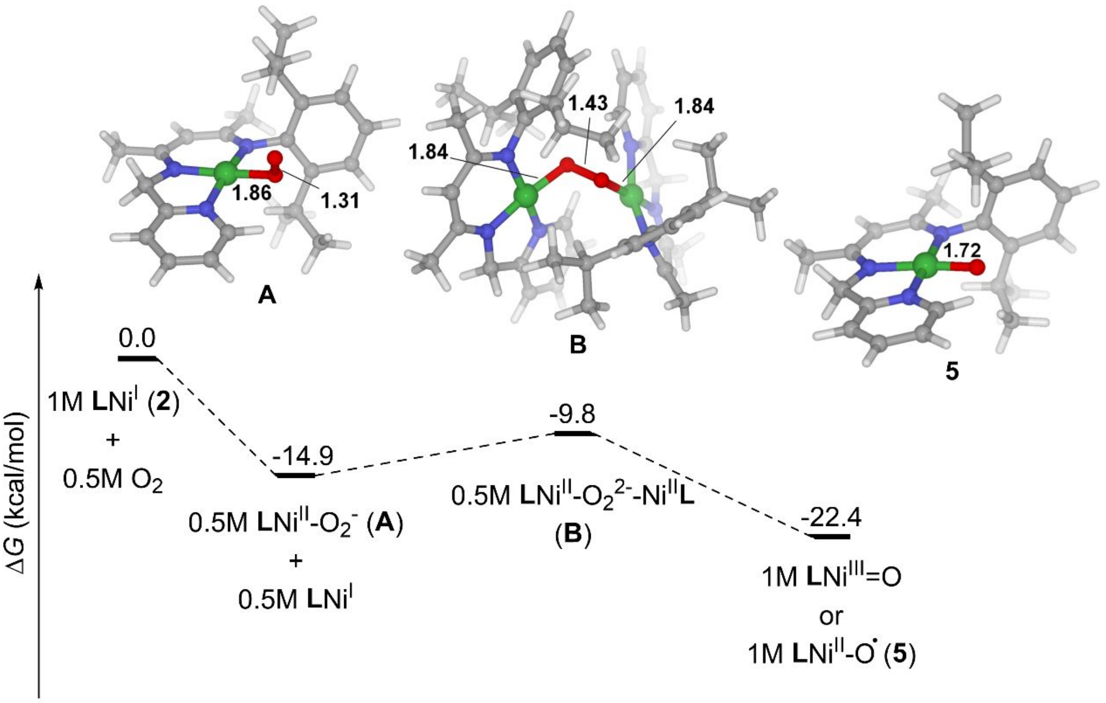

Figure S9A. SMD-TPSSh/Def2-TZVP//SMD-TPSS/Def2-SVP free energy profile of the conversion of **2** to **5**. The optimized structures of the proposed Ni-η^1-superoxo and Ni_2-μ-1,2-peroxo intermediates are given (gray: carbon, blue: nitrogen, green: nickel, red: oxygen). The unit of energy is kcal/mol.

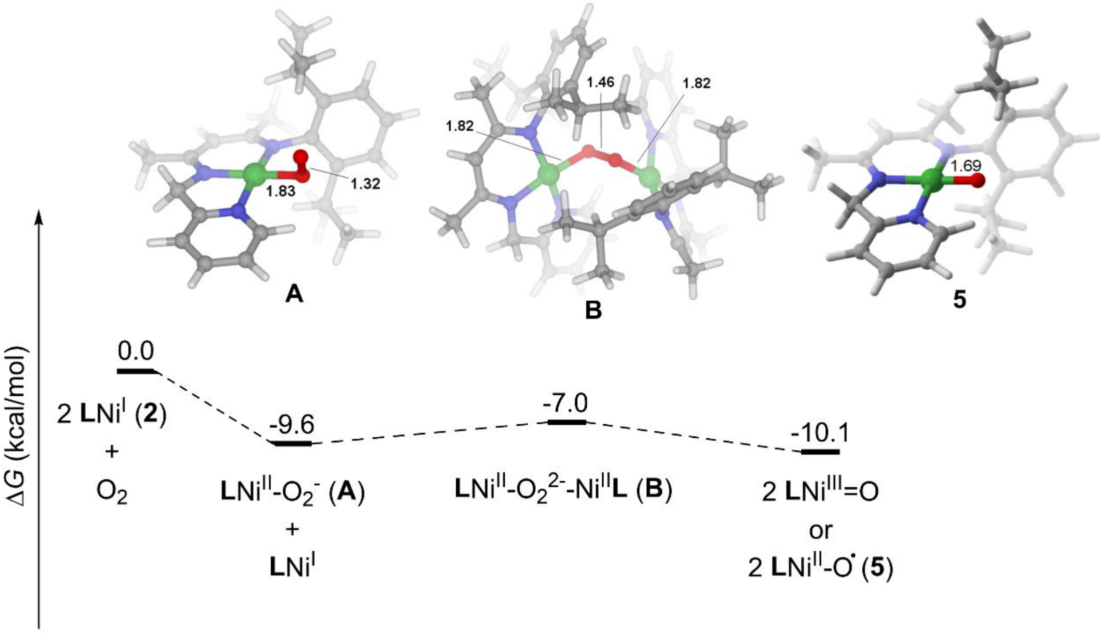

Figure S9B. SMD-B97/6-311+G(d,p)//B97/6-31G(d) free energy profile of the conversion of **2** to **5**. The optimized structures of the proposed Ni-η^1-superoxo and Ni_2-μ-1,2-peroxo intermediates are given (gray: carbon, blue: nitrogen, green: nickel, red: oxygen). The unit of energy is kcal/mol.

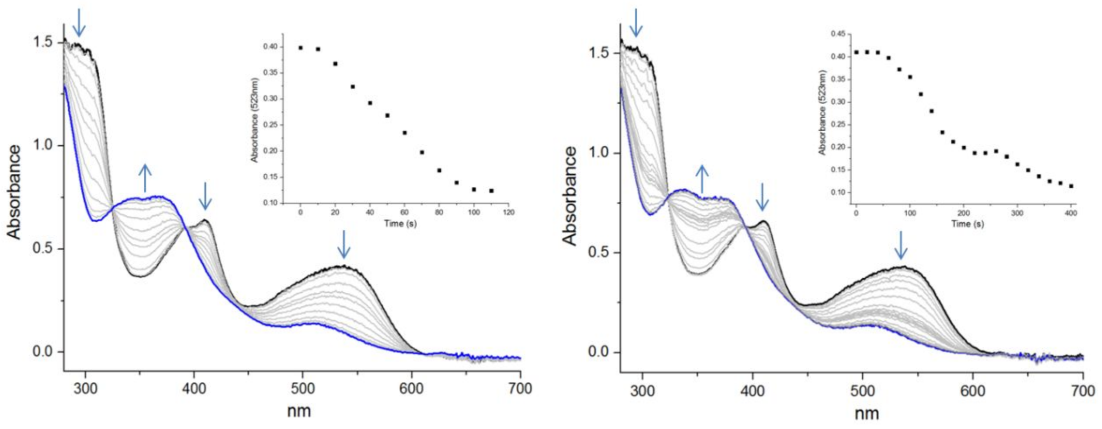

Figure S10. UV–vis spectral changes accompanying oxygenation of **2** (0.15 mM) in MTHF at -80 ˚C (left) and -110 ˚C (right), recorded every 20 s. The initial and final spectra are represented in black and blue, respectively. The band at 523 nm decreases steadily over time. Inset: kinetic trace at 523 nm.

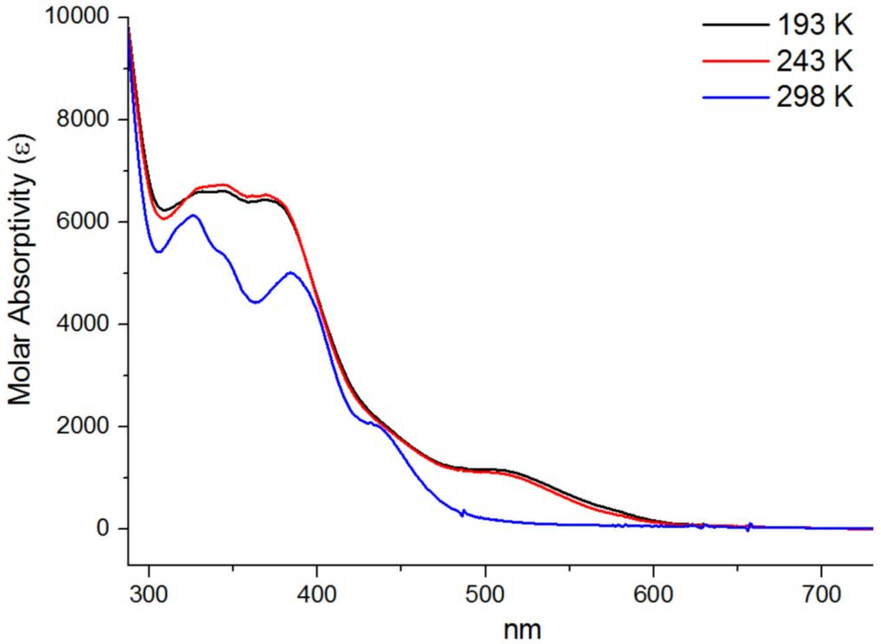

Figure S11. Electronic spectrum in Toluene of **5** (black line) at 193K, **5** (red line) at 243 K, and **4** (blue line) at 298K.

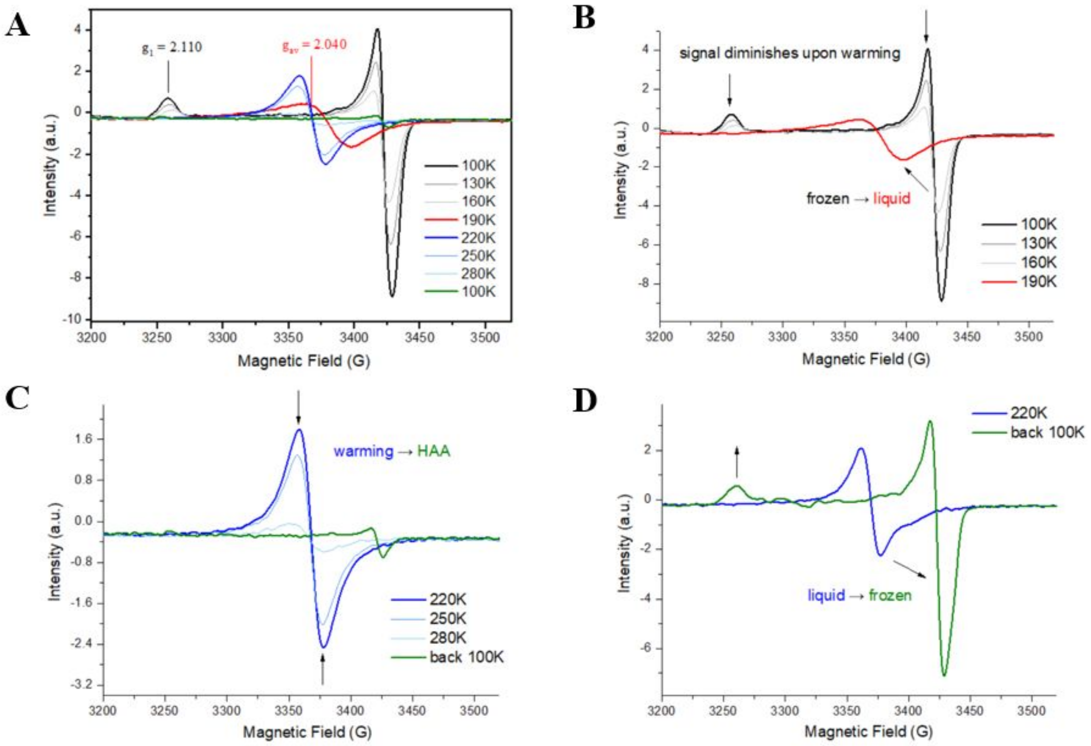

Figure S12. (A) EPR spectra of an *in-situ* generated **5** monitored during the temperature change from 100 K (black line) to 280 K (blue line) back to 100 K (green line) in toluene. (B) Frozen-to-mobile transition upon warming (100–190 K). (C) Signal decay at higher temperature (220–280 K) due to HAA, with no recovery upon cooling. (D) Spectrum obtained upon cooling from 220 K back to 100 K. (microwave frequency: 9.628 GHz, microwave power: 0.32 mW).

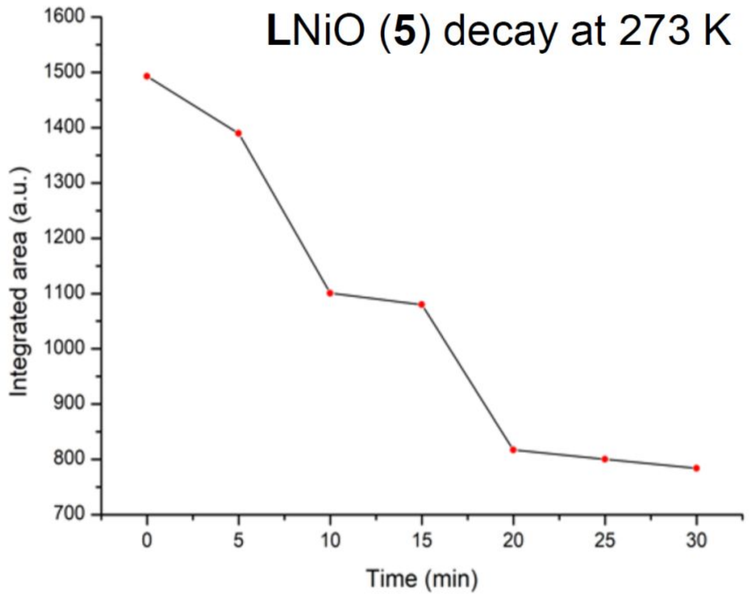

Figure S13. Degradation of complex **5** by EPR spectrum monitoring over time at a temperature of 273 K in toluene (microwave frequency: 9.628 GHz, microwave

power: 0.32 mW).

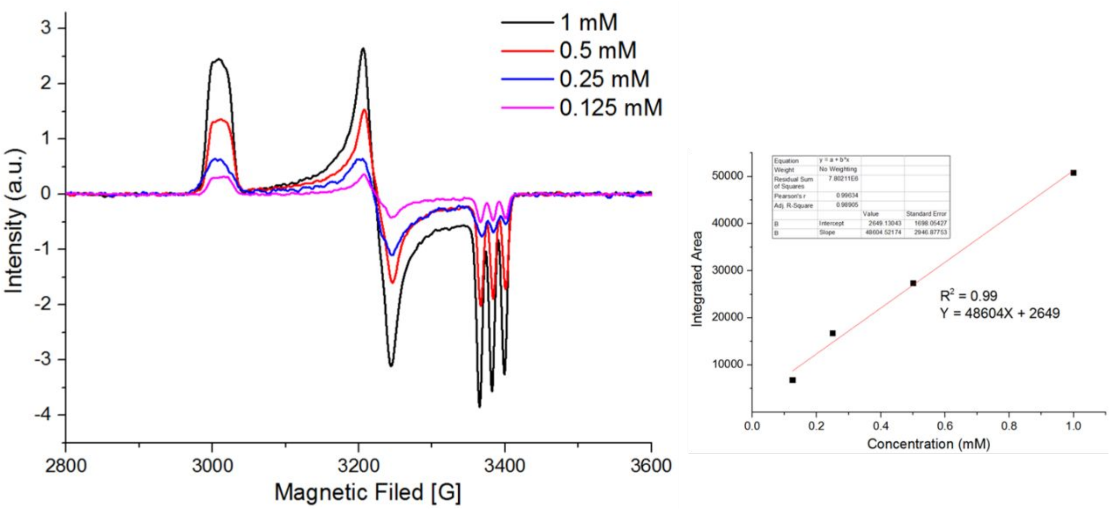

Figure S14A. EPR spectra of **2** at different concentrations showing linear intensity increase with concentration (inset, R² = 0.99) in toluene at 100K (microwave frequency: 9.628 GHz, microwave power: 0.32 mW).

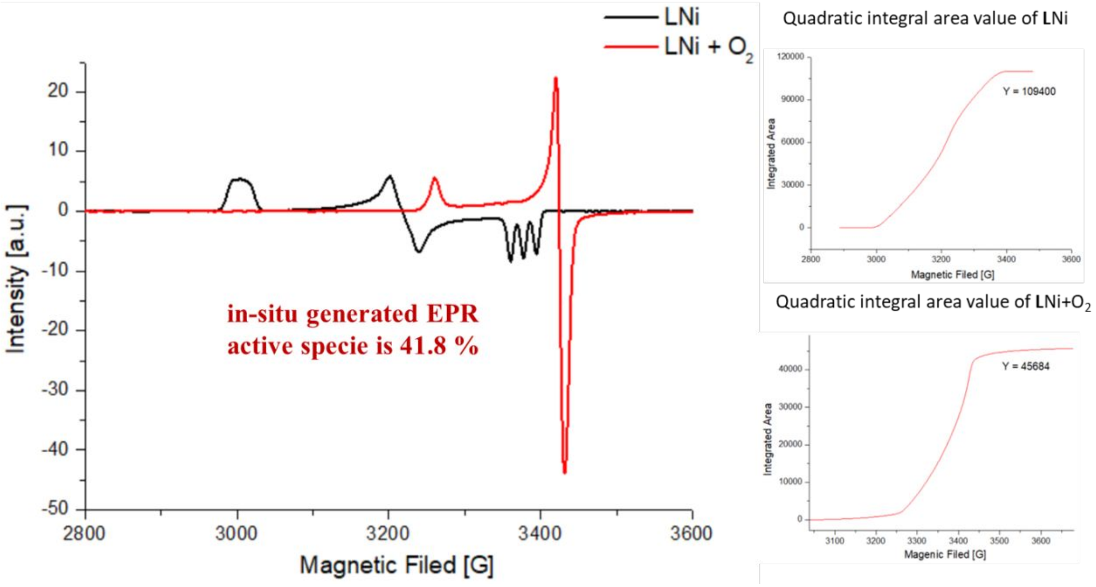

Figure S14B. EPR spectra of **2** (black line) and **5** generation by **2** (2.2 mM) react with O_2 (red line) in toluene at 100K (microwave frequency: 9.628 GHz, microwave power: 0.32 mW).

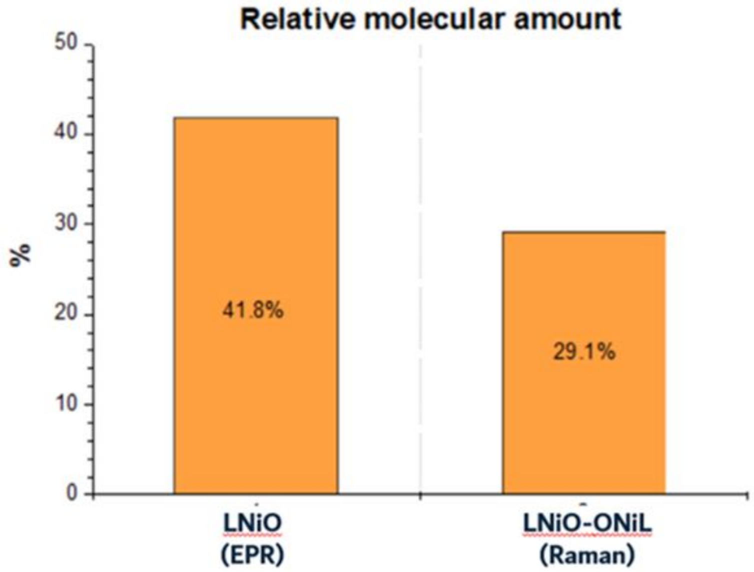

Figure S14C. Relative molecular distribution of Ni–O species generated from the reaction of **L**Ni^{I} (**2**) with O_2. EPR analysis reveals 41.8% of the paramagnetic **L**NiO species (**5**), while Raman spectroscopy identifies 29.1% of the μ-peroxo dimer (**L**NiO–ONi**L**; **B**).

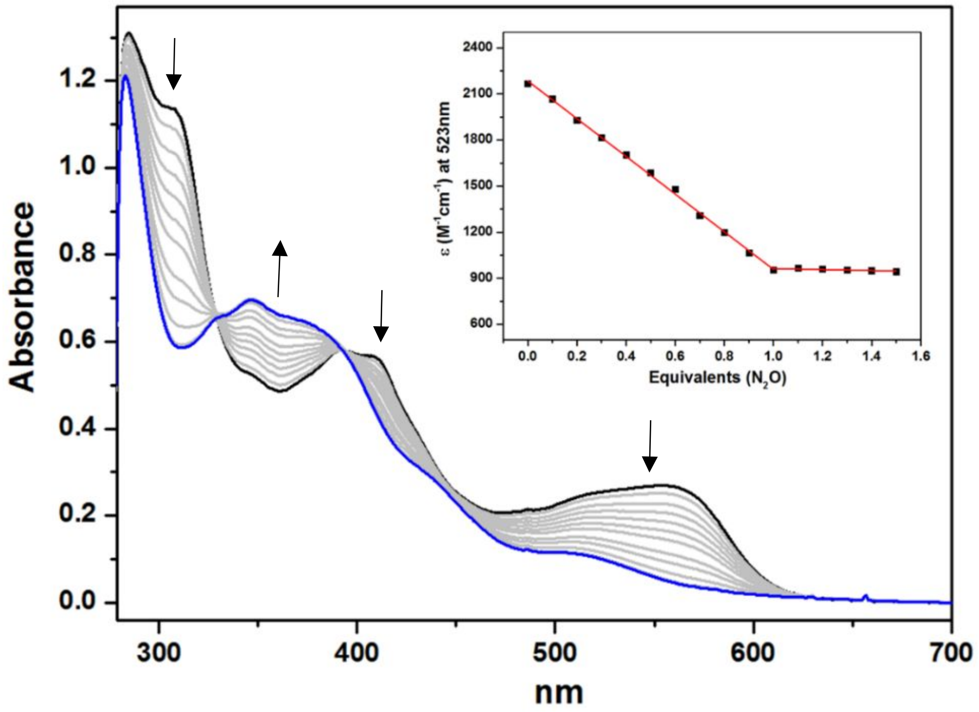

Figure S15A. UV-vis changes accompanying the oxygenation of **2** in toluene at -80 °C, using initial concentrations of 0.12 mM. The 0.1 equivalent of N_2O was added by 5-minute increments until 1.0 equivalent. The initial and final spectra are represented in black and blue, respectively. The insets show the results of spectrophotometric titrations of complex **2** with N_2O at 523 nm, plotted as linear fits at 1:1 Ni/N_2O ratio.

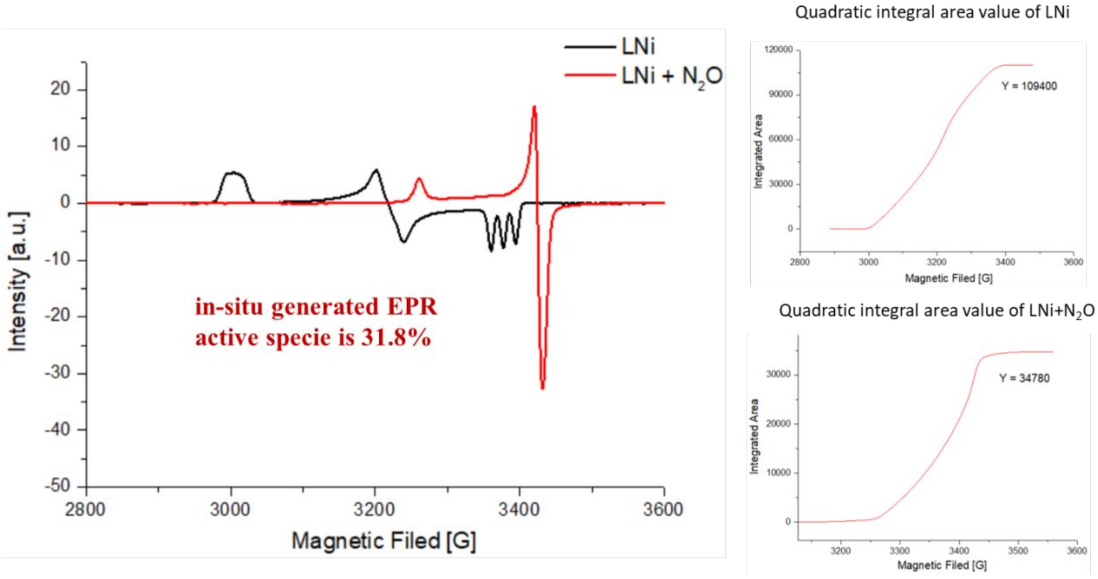

Figure S15B. EPR spectra of **2** (black line) and **5** generated by **2** (2.2 mM) with N_2O (red line) in toluene at 100K (microwave frequency: 9.628 GHz, microwave power: 0.32 mW).

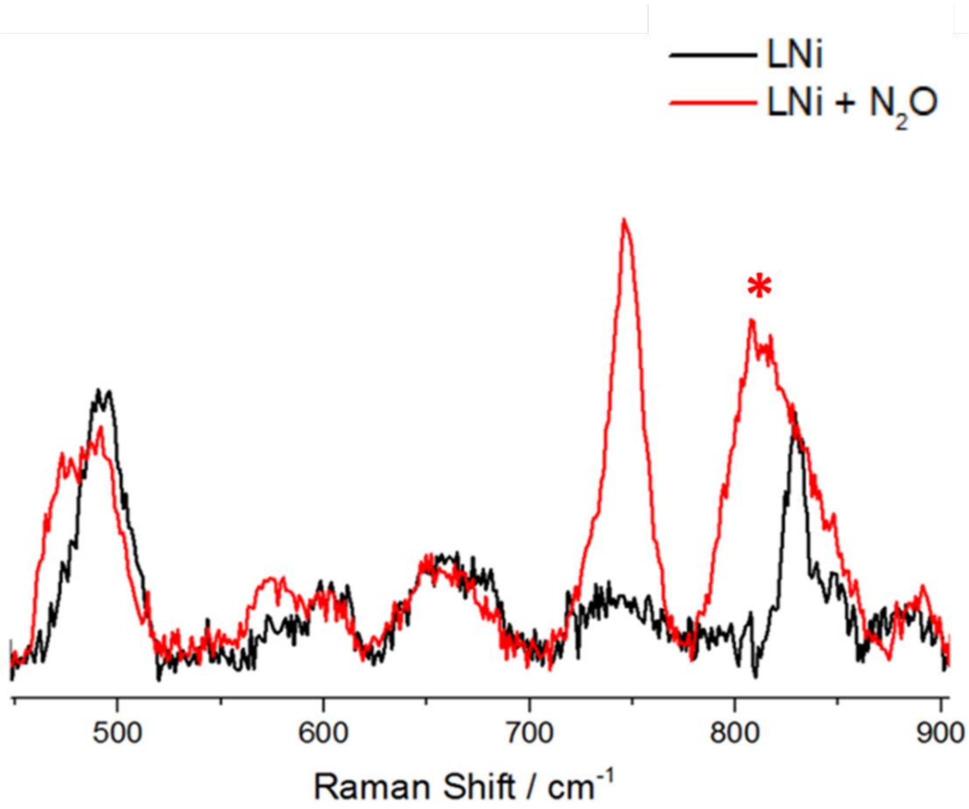

Figure S15C. Raman spectra (λ_{ex} = 532 nm) of **2** (black line) and **5** generated by **2** with N_2O (red line) in MTHF at -80 °C (*denotes solvent peak).

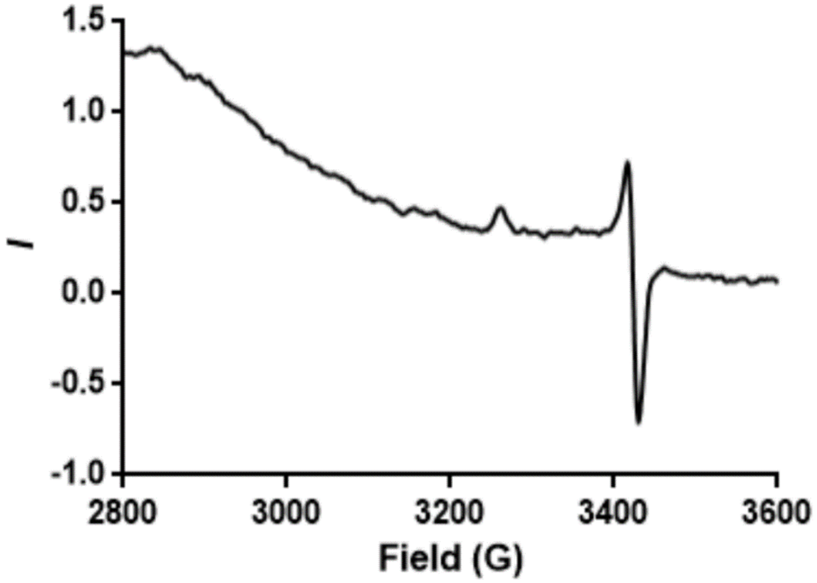

Figure S16A. EPR spectrum of **5** generation by **2** react with TMAO in THF at 100 K (microwave frequency: 9.628 GHz, microwave power: 0.32 mW).

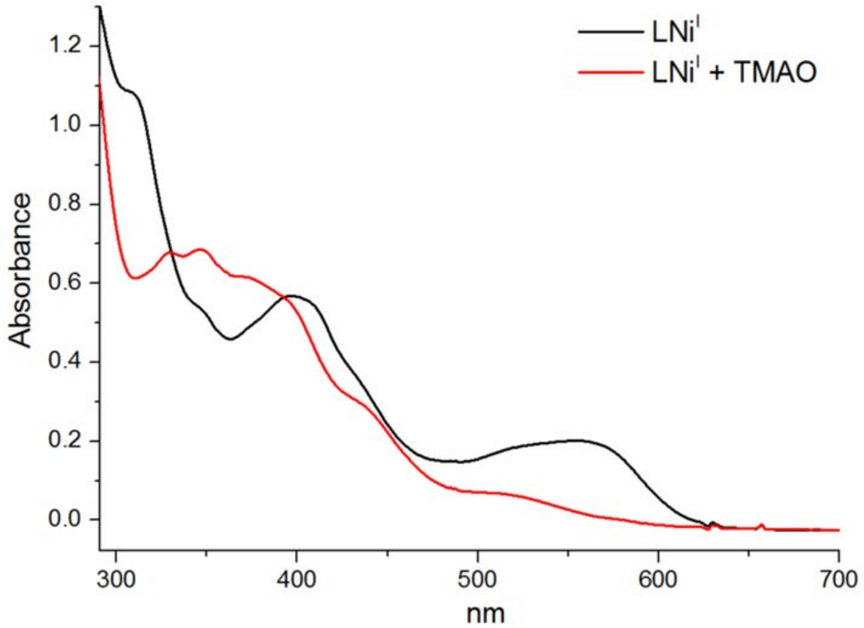

Figure S16B. UV spectrum of **5** generation by **2** react with TMAO in toluene at -80° C.

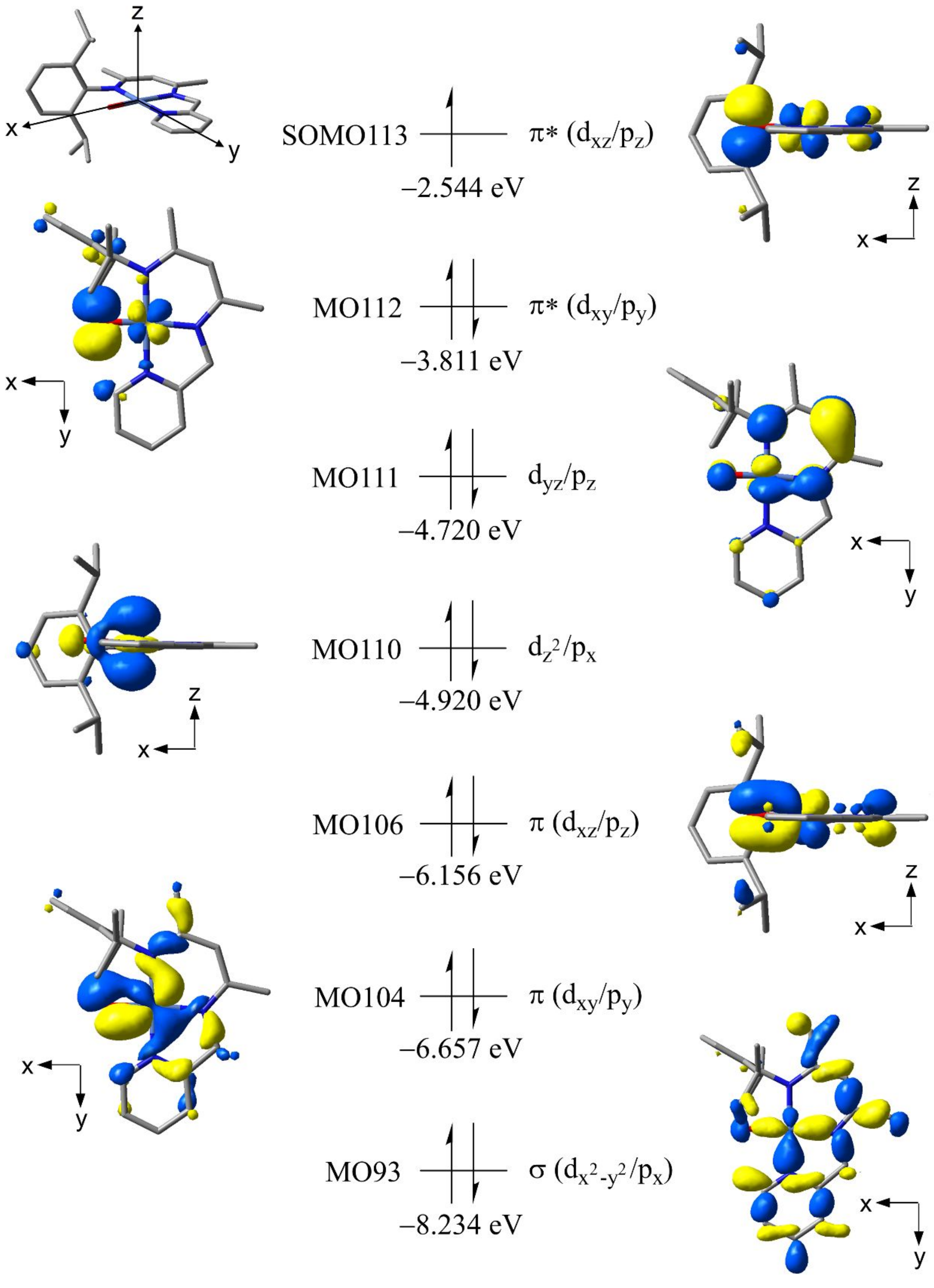

Figure S17. Selected molecular orbitals of the restricted open-shell wavefunction of the unsymmetrical β-diketiminato nickel-monooxygen complex **5**.

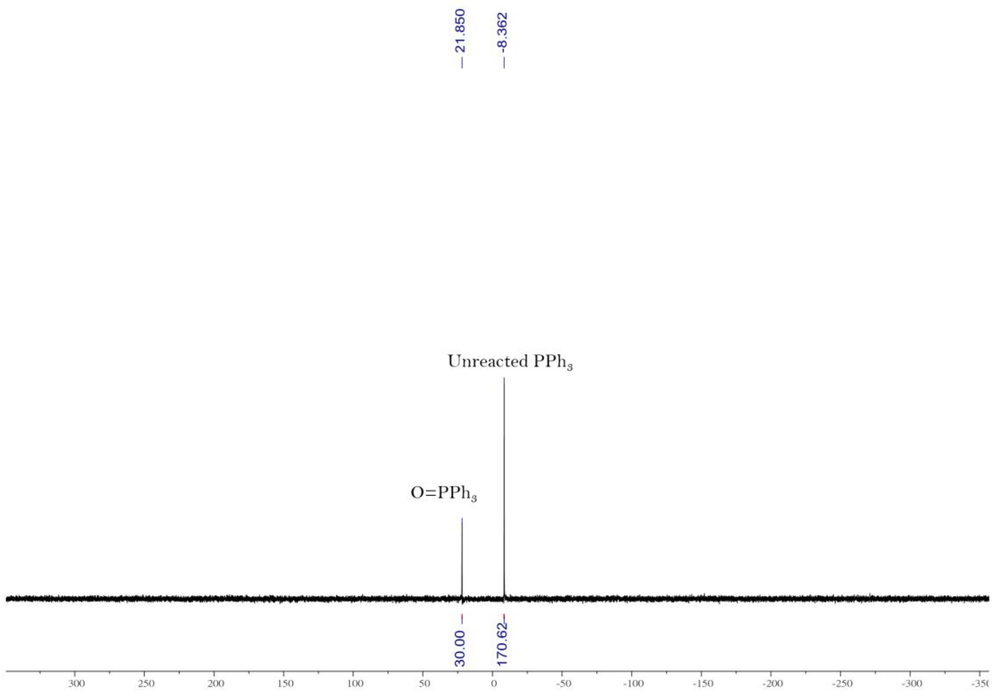

Figure S18. ^{31}P NMR spectrum of **5** react with PPh_3(two equivalences) in C_6D_6 (121 MHz, 298 K).

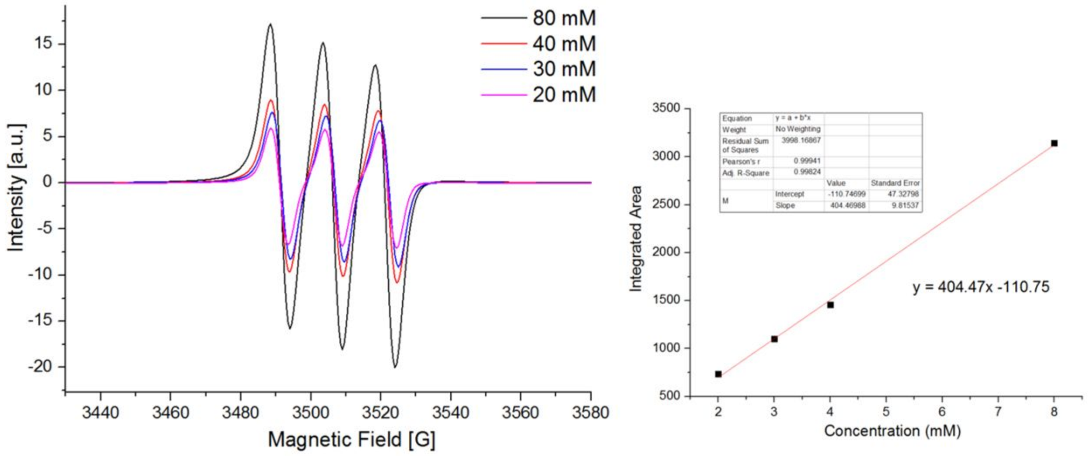

Figure S19A. EPR spectra of TEMPO▪ at varying concentrations showing linear intensity increase with concentration (inset, $R^2 = 0.99$) in toluene at 298K (microwave frequency: 9.64 GHz, microwave power: 10 mW).

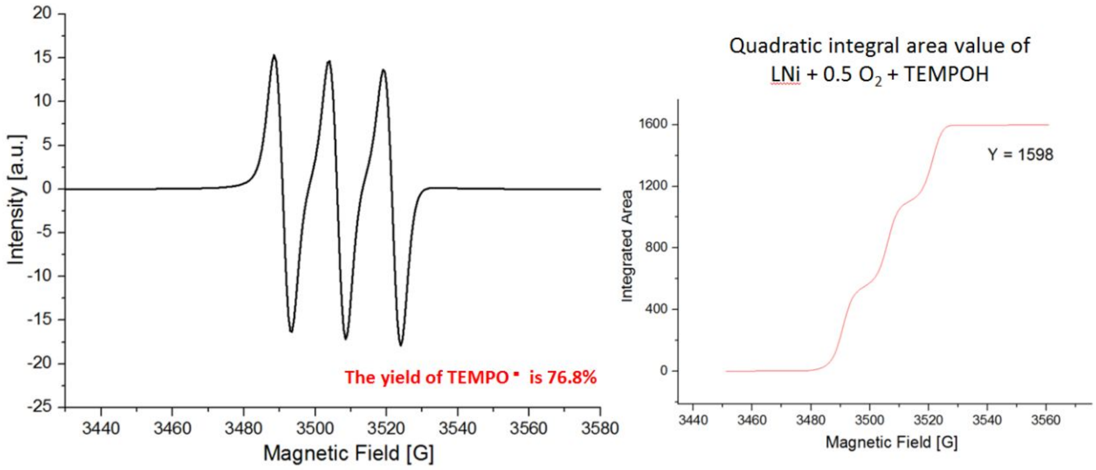

Figure S19B. EPR spectrum (298 K) of **5**, generated in situ from **2** and O_2 in toluene at 193 K, followed by reaction with 1 equiv of TEMPOH (5.5mM) in toluene at 243 K overnight (microwave frequency: 9.64 GHz, microwave power: 10 mW).

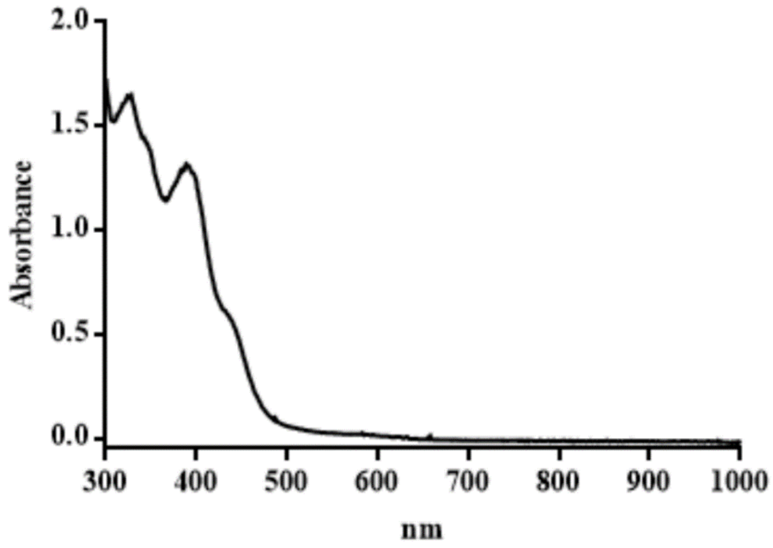

Figure S20. Electronic spectrum in toluene after **5** react with TEMPOH at 193 K and warmed to 298K.

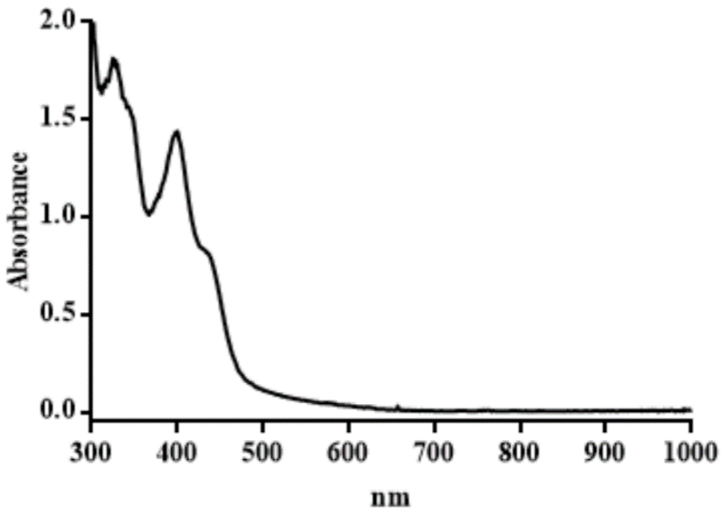

Figure S21. Electronic spectrum in toluene after **4** react with TEMPO at 298K.

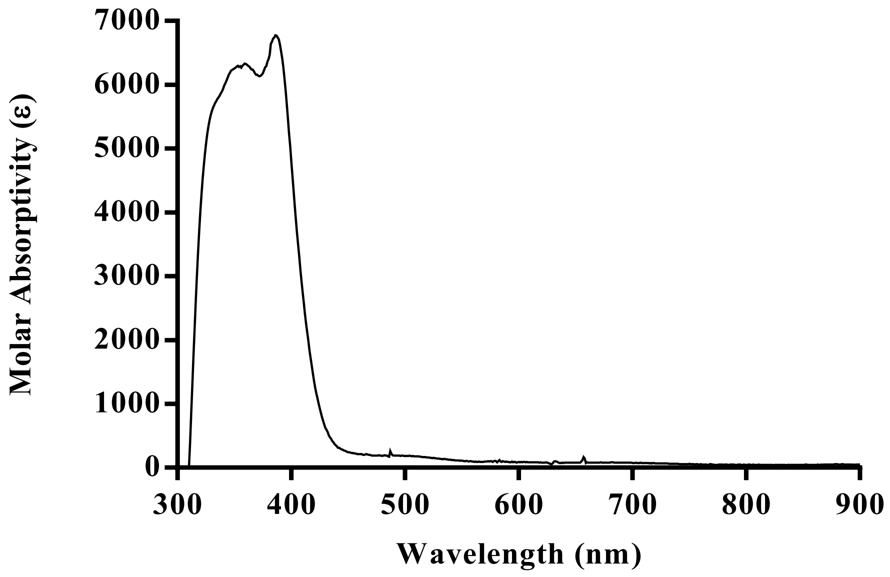

Figure S22. Electronic spectrum of **1** in toluene at 298 K

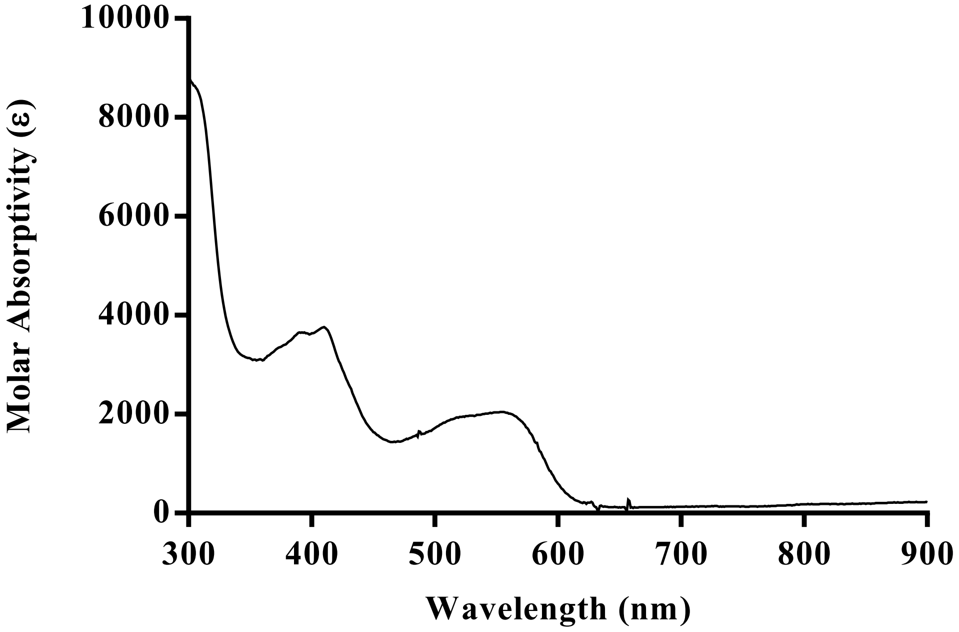

Figure S23. Electronic spectrum of **2** in toluene at 298 K.

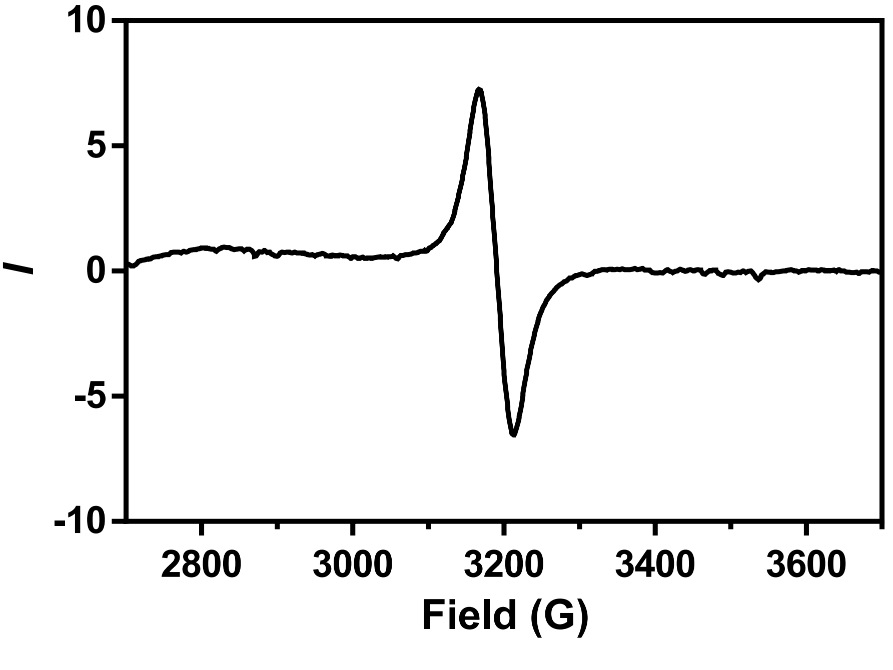

Figure S24. EPR spectrum of **2** in toluene at 298 K (microwave frequency: 9.64 GHz, microwave power: 10 mW).

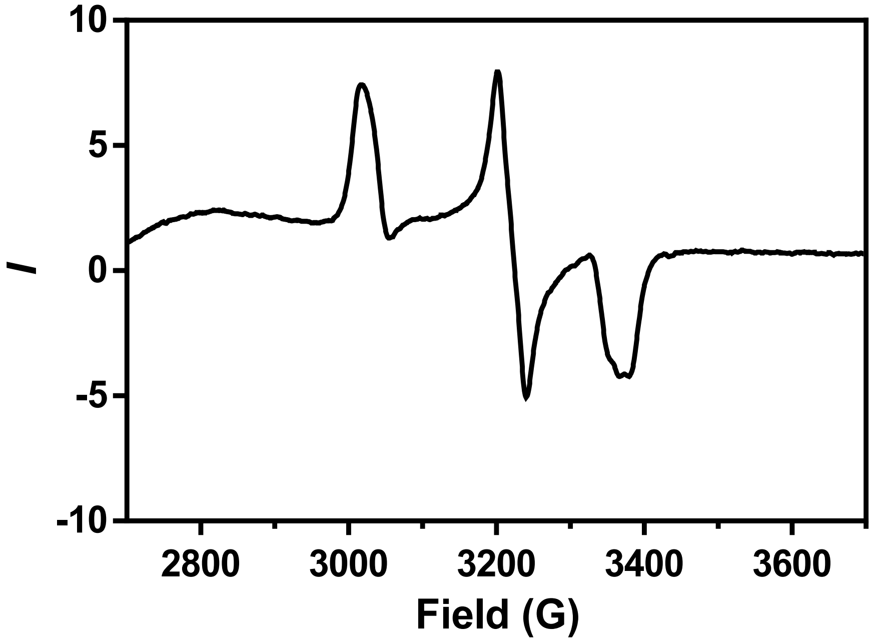

Figure S25. EPR spectrum of **2** in toluene at 193 K (microwave frequency: 9.64 GHz, microwave power: 10 mW).

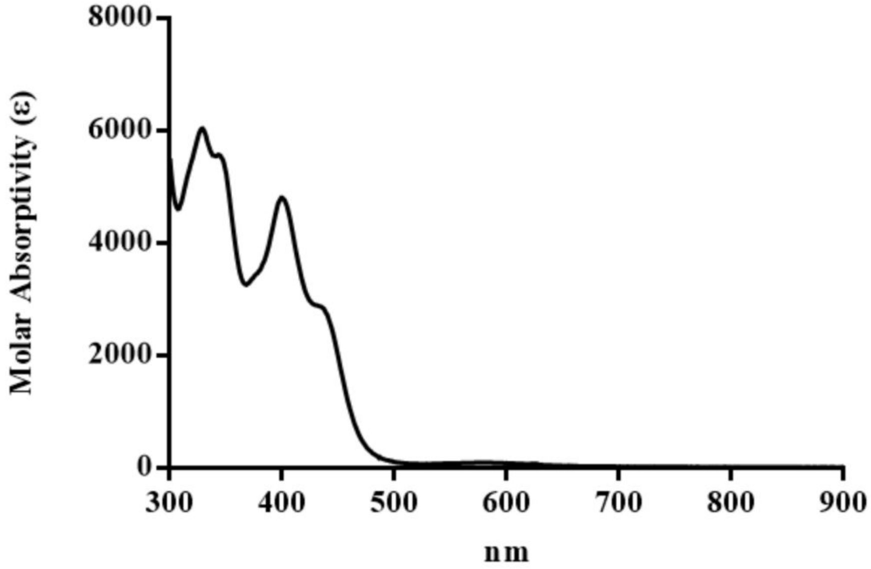

Figure S26. Electronic spectrum of **4** in toluene at 298 K

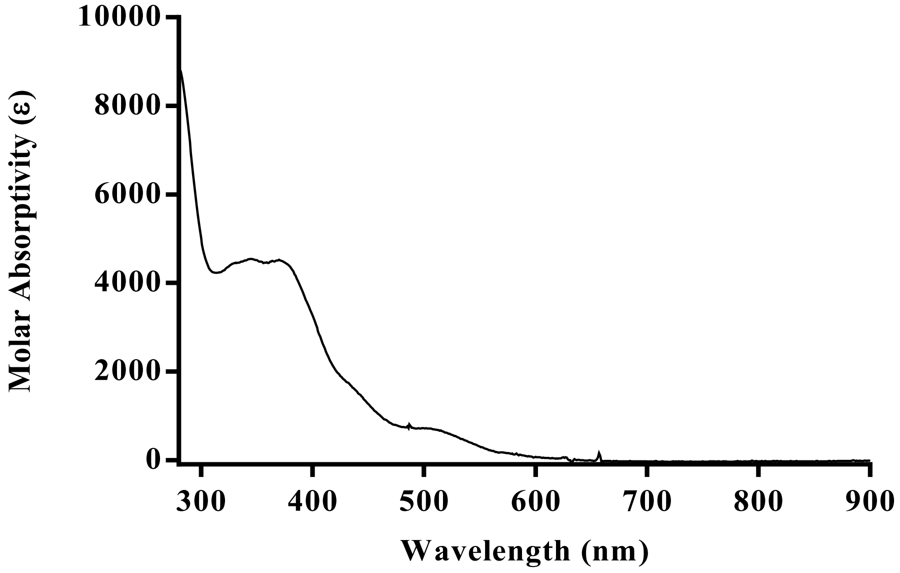

Figure S27. Electronic spectrum of **5** in toluene at 193 K.

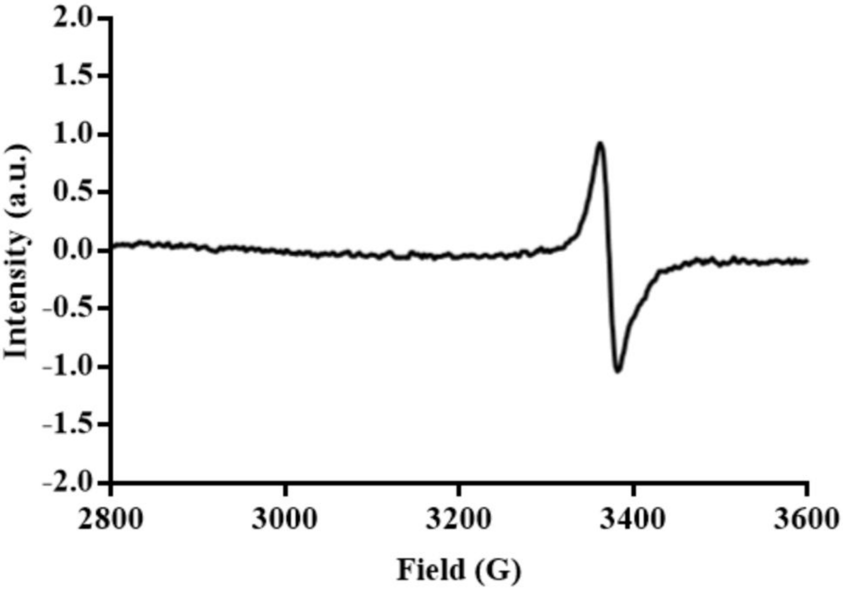

Figure S28. EPR spectrum of **5** in toluene solution at 243 K (microwave frequency: 9.64 GHz, microwave power: 10 mW).

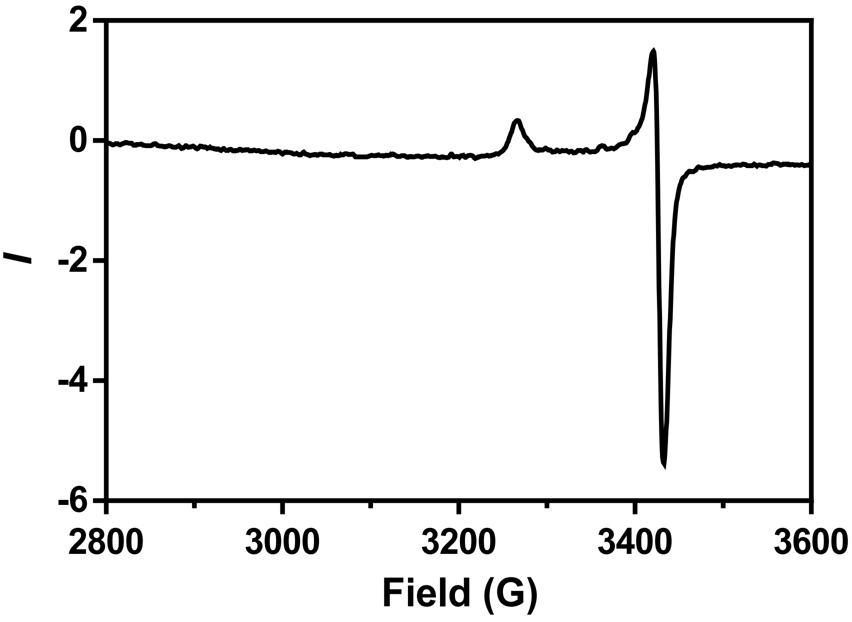

Figure S29. EPR spectrum of **5** in toluene solution at 108 K (microwave frequency: 9.64 GHz, microwave power: 10 mW).

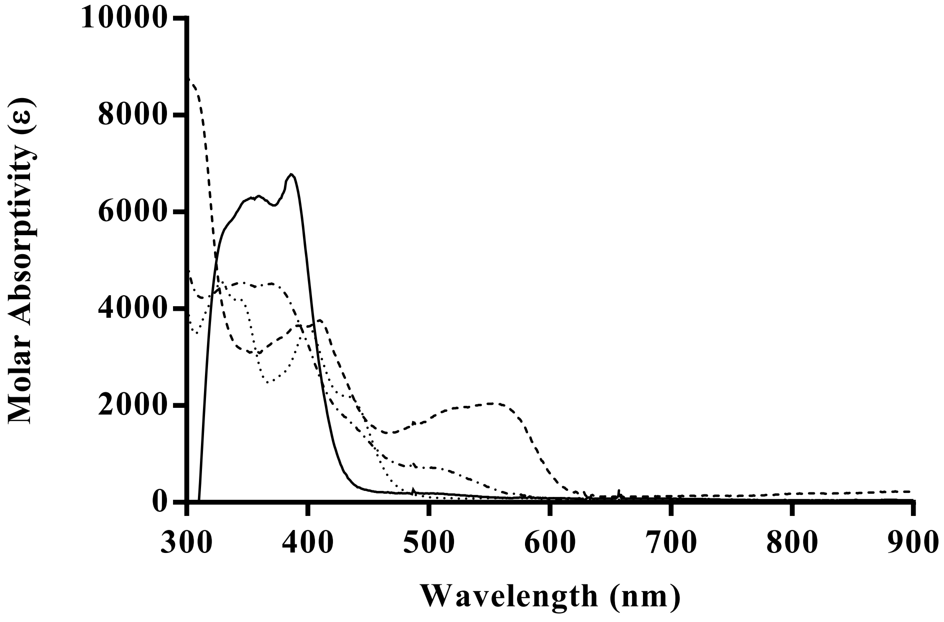

Figure S30. Electronic spectrum of **1** (solid line), **2** (dashed line), **4** (dotted line), and **5** (dashed-dot line) in toluene at 193 K.

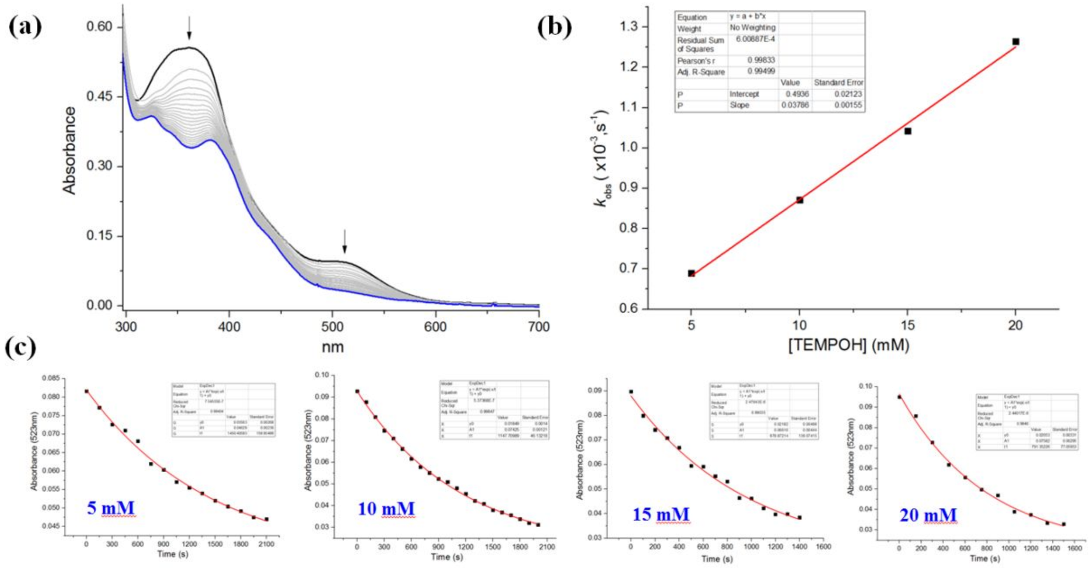

Figure S31. (a) UV–vis kinetic spectra for the reaction of **5** (0.125 mM), generated in situ from **2** and O_2 in toluene at −35 ˚C, with TEMPOH (10 mM). Initial: black line; final: blue line. (b) Plot of k_{obs} versus the concentration of TEMPOH. (c) Time-dependent absorbance plots at 523 nm with varying concentrations.

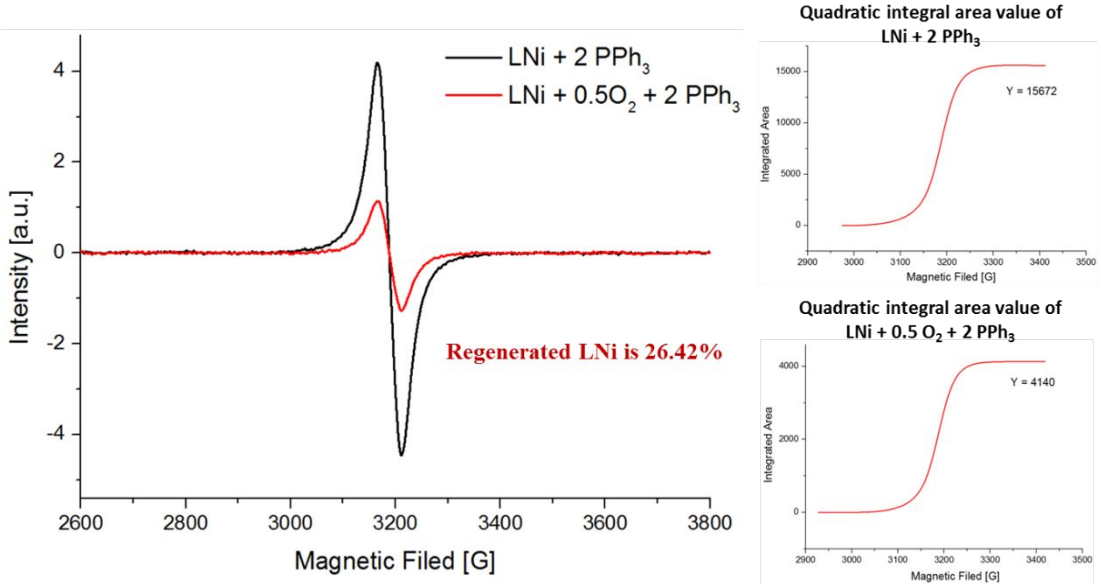

Figure S32. X-band EPR spectra of **2** with 2 equiv PPh_3 (black) and in-situ prepared **5** followed by 2 equiv PPh_3 at 243K (red), recorded in toluene at 298 K. (microwave frequency: 9.64 GHz, microwave power: 10 mW).

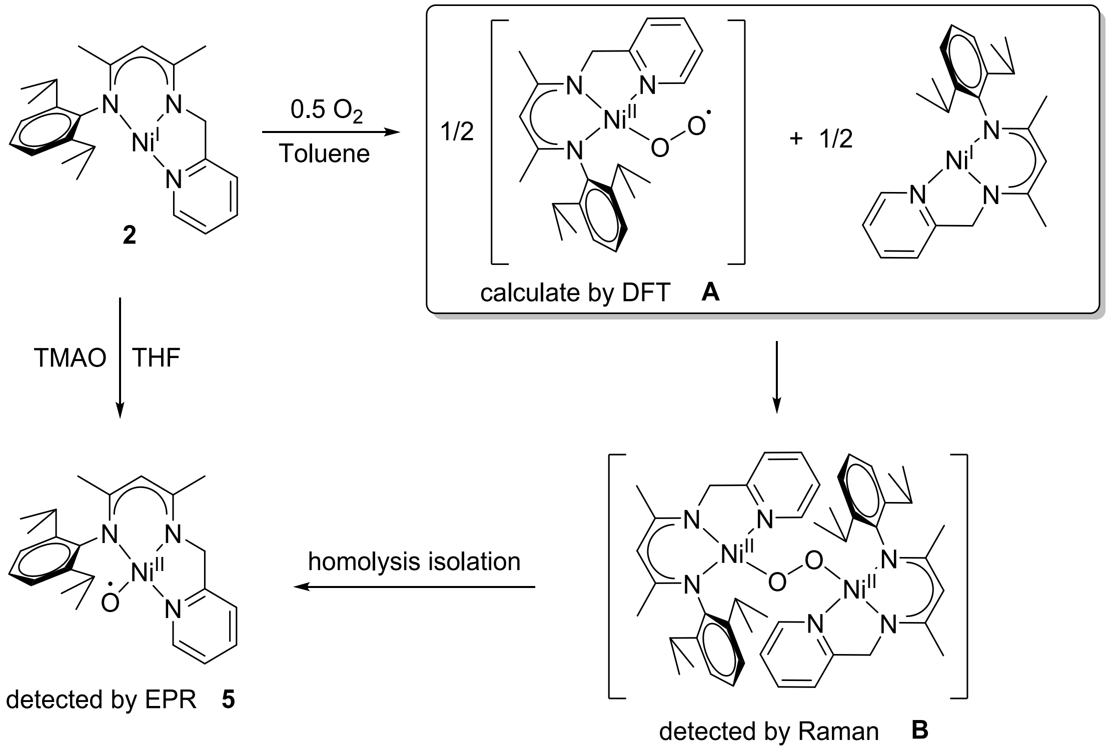

Scheme S1. Representation of a nickel-oxygen from the *N*-aryl-*N*′-methylpyridyl β-diketiminato nickel(I) complex with two different oxygen sources and their pathway.

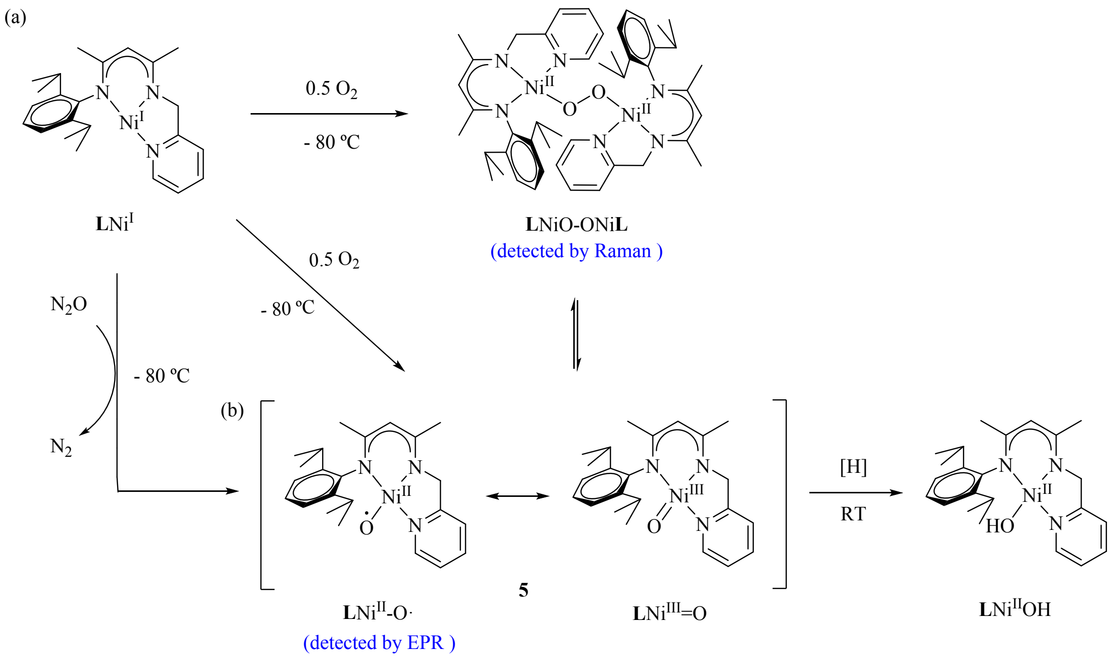

Scheme S2. (a) Synthesis of the *N*-aryl-*N*′-methylpyridyl β-diketiminato nickel-oxygen complexes. (b) Representations of **5** as a Ni^{III}=O or Ni^{II}–$O^{\bullet}$ species.
